# Evaluating Plasma‐Handling Effects on Extracellular Vesicle RNA Yield and Cargo Profiles in Fish: A Methodological Insight

**DOI:** 10.1002/jex2.70175

**Published:** 2026-10-15

**Authors:** Antonio Espuela‐Ortiz, Estrella Antón‐González, Louis Bonnefous, M. Virginia Martín, Salvador Jeréz, Deiene Rodríguez‐Barreto

**Affiliations:** ^1^ Department of Animal Biology, Soil Science and Geology Universidad de La Laguna San Cristóbal de La Laguna Tenerife Spain; ^2^ Canary Islands Oceanographic Center (COC) IEO‐CSIC Santa Cruz de Tenerife Spain

**Keywords:** exoRNeasy, extracellular vesicle, freeze‐thaw, gilthead sea bream, kit format, MinION Mk1C, Oxford Nanopore Technology, plasma, *Sparus aurata*, teleost fish

## Abstract

Extracellular vesicle (EV)‐derived RNA (EV‐RNA) is emerging as a valuable biomarker source in aquatic species. However, the composition and stability of long RNA species, particularly messenger RNA (mRNAs) and long non‐coding RNAs (lncRNAs), remain largely unexplored in teleosts. Pre‐analytical factors such as input plasma volume and storage conditions may influence RNA recovery, especially in low‐input samples like fish plasma. Here, we evaluated the effects of input sample volume and freezing storage on EV‐RNA yield and integrity in gilthead sea bream (*Sparus aurata)* plasma using the exoRNeasy commercial kit. Both Maxi (∼2 mL) and Midi (∼1 mL) kit formats produced comparable RNA recovery, indicating flexibility in plasma volume requirements. Freezing did not alter the overall RNA biotype composition or the identity of the dominant transcripts but resulted in a higher proportion of shorter and incomplete isoforms, suggesting selective susceptibility to degradation of long RNAs during freezing storage.

Using Oxford Nanopore Technologies long‐read sequencing, we generated the first long RNA‐resolved EV transcriptome for this species. EV cargo was dominated by ribosomal and metabolic mRNAs, consistent with conserved vertebrate EV signatures, and 302 distinct lncRNAs were identified, two of which accounted for nearly 90% of lncRNA reads. Predicted interactions between abundant lncRNAs, EV mRNAs, and their encoded ribonucleoproteins suggest coordinated packaging of RNA–protein modules.

Overall, this study validates exoRNeasy for fish plasma and provides the first comprehensive long‐RNA EV landscape in a teleost, providing a valuable framework for future molecular and functional studies in non‐model fish species.

## Graphical abstract

Experimental Design Overview

Illustration to visualize the experimental structure (can be included in a graphical abstract later on)—Diagram / Workflow showing fish plasma collection, sample conditions: (a) volume groups (1 mL vs 2 mL or Midi vs Maxi); (b) Fresh vs frozen EV isolation→RNA extraction→Sequencing pipeline.

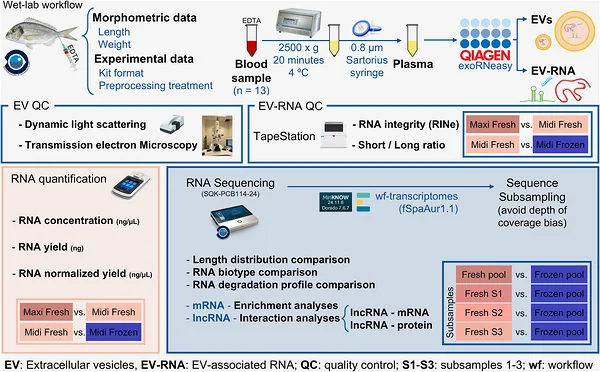

## 1 Introduction

Extracellular vesicles (EVs) are membrane‐bound nanoparticles secreted by virtually all cell types and are increasingly recognized as key mediators of intercellular communication. They carry a wide variety of bioactive molecules (e.g., proteins, lipids, and nucleic acids) that can be transferred between cells, influencing gene expression, metabolism, and immune responses (Baj‐Krzyworzeka et al. 2002, Baj‐Krzyworzeka et al. 2007; Kemunto et al. 2025; Kumar et al. 2024; Wysoczynski and Ratajczak 2009). The molecular content of EVs may also reflect the physiological state of their parent cells (Schorey et al. 2015; Szatanek et al. 2017). Through this cargo transfer, EVs participate in essential physiological processes and are now considered important components of intercellular signaling networks in both health and disease (Kumar et al. 2024). Moreover, EVs have been proposed to mediate communication between somatic and germline cells, potentially contributing to the transmission of epigenetic information to the offspring (Spadafora 2020).

Research on EVs has grown exponentially in mammalian systems (Bazzan et al. 2021; Couch et al. 2021), revealing the diagnostic and therapeutic potential of their molecular cargo, particularly small RNAs such as microRNAs (miRNAs) (Kumar et al. 2024; O'Brien et al. 2020; Xu et al. 2022). However, in comparison to small RNA biotypes, long RNA biotypes (i.e. messenger RNAs [mRNAs] and long non‐coding RNAs [lncRNAs]) remain less thoroughly characterized, although research in this area is expanding rapidly. These long RNAs extend the regulatory landscape of EVs, potentially mediating transcriptional and translational control, chromatin remodelling, and post‐transcriptional regulation in recipient cells (Fabbiano et al. 2020; Statello et al. 2018). While their presence has been documented across multiple mammalian models, their stability, packaging mechanisms, and biological functions are still not fully understood (O'Grady et al. 2022).

In aquatic species, EV research is still at an early stage (Zhao, Deng et al. 2021). Fish represent an emerging model for EV biology due to their relevance in aquaculture and their physiological adaptability to environmental challenges. Several studies have demonstrated the presence of EVs in fish plasma, mucus and reproductive fluids, confirming morphological and biochemical features consistent with those of mammalian EVs (Nikapitiya et al. 2022; Pelyhe and Sturve 2023; Zhao et al. 2020). In teleost fish, the characterization of the RNA cargo has mainly focused on small RNAs, including miRNAs and Piwi‐interacting RNAs (piRNAs) (Magnadóttir et al. 2020; Pan et al. 2025; Smith et al. 2020; Zhao et al. 2020; Zhao, Zhang et al. 2021) and on protein cargo (Zhao, Deng et al. 2021). Nevertheless, the characterization of the long RNA fraction of EV cargo, especially the lncRNA biotype, remains scarce. Understanding EV‐RNA content in fish could provide insights into systemic communication under stress, infection, or metabolic regulation, processes of high importance in aquaculture and environmental physiology.

EVs can be isolated from diverse biological sources using multiple methods, but no gold‐standard protocol exists to date (Brennan et al. 2020). Differential centrifugation remains the most widely used technique, though it may cause EV loss or damage, and often co‐isolates contaminants (Hessvik and Llorente 2018; Momen‐Heravi 2017; Théry et al., 2006). Alternative approaches, including polymer‐based precipitation (Ryu et al. 2020), density‐gradient separation (Kowal et al. 2016; Onódi et al. 2018), size‐exclusion chromatography (An et al. 2018; Böing et al. 2014), immunoaffinity capture (Filipović et al. 2022; Greening et al. 2015; Tauro et al. 2012), and various commercial kits, offer different balances of purity, yield and practicality. EV and EV‐associated RNA (EV‐RNA) isolation can be carried out independently or concatenated, and often require lengthy workflows, specialized equipment or the concatenation of different methods to overcome their limitations. Performing EV and EV‐RNA isolation simultaneously might avoid EV degradation and reduce EV‐RNA degradation due to sample handling and storage. Membrane‐based platforms such as the ExoRNeasy kit streamline the workflow by integrating EV capture with direct EV‐RNA extraction, providing a fast, reproducible, and standardized alternative that avoids the complexity, variability and co‐isolation issues associated with traditional EV enrichment, though at the expense of EV specificity (Enderle et al. 2015; Gutiérrez García et al. 2020).

Despite significant methodological advances, pre‐analytical variables such as sample volume, storage temperature, freeze‐thaw cycles, handling procedures and RNA isolation method can significantly influence the yield, integrity and bias the RNA composition isolated from EVs (Ahmadian et al. 2024; Buschmann et al. 2018; Enderle et al. 2015). Plasma and serum are among the most frequently used biofluids for EV research, yet the heterogeneity in EV yield and RNA composition across protocols continues to hinder reproducibility and cross‐study comparisons (Enderle et al. 2015; Théry et al. 2018). Understanding and mitigating these effects is essential for developing reliable and comparable methodologies for EV‐RNA research in fish, where standardization and systematic evaluation of pre‐analytical effects is lacking.

Studies using human and animal plasma have shown that RNA yield from EVs scales non‐linearly with input volume (Enderle et al. 2015; Helwa et al. 2017; Théry et al. 2018). Enderle et al. (2015) showed that RNA recovery increases proportionally from 0.5 mL up to 4 mL of plasma, while it may plateau or even decrease in efficiency at larger input volumes, likely due to column saturation or co‐isolation of non‐vesicular material (Enderle et al. 2015). Such discrepancies underline the importance of optimizing the extraction volume for each biological source. For low‐biomass samples, such as fish plasma, where available volumes are typically below 2 mL, understanding volume‐dependent effects is essential to balance yield and purity. While manufacturers of commercial EV‐RNA extraction kits (e.g., exoRNeasy, Qiagen) report comparable efficiency across different kit formats, independent validation in non‐mammalian species is lacking.

Storage temperature and duration represent another major source of variability in EV and EV‐RNA studies. Mammalian data indicate that freezing and thawing cycles can compromise vesicle integrity, promote EV aggregation, and affect RNA yield or bias their composition (Ahmadian et al. 2024; Gelibter et al. 2022; Tessier et al. 2021). Nonetheless, findings are inconsistent: some studies report significant RNA loss or fragmentation after prolonged storage, particularly at –20°C, while others find minimal impact when samples are rapidly frozen and stored at –80°C (Ahmadian et al. 2024). For instance, Enderle et al. (2015) observed stable miRNA and mRNA recovery from serum EVs stored at –80°C for several months (Enderle et al. 2015), whereas Gelibter et al. (2022) documented reduced RNA quantity and increased degradation in low‐biomass plasma samples subjected to similar conditions (Gelibter et al. 2022). These discrepancies likely reflect differences in isolation methods and input plasma volumes, as well as inherent sample variability.

Moreover, most of the studies have focused on small RNAs, while the impact of freezing on long RNA integrity, isoform completeness, and transcript‐length distribution remains largely unexplored. Long RNAs are generally more susceptible to degradation, and subtle losses may not be detected by bulk quantification but could affect downstream transcriptomic analyses, particularly those aiming to resolve full‐length mRNAs or lncRNAs. Thus, assessing how pre‐analytical handling influences long RNA recovery is crucial to establish reliable workflows for EV‐RNA studies.

Given these challenges, there is a clear need to validate standardized protocols for EV‐RNA isolation and quality control in non‐mammalian species. Gilthead sea bream (*Sparus aurata*) represents a valuable model due to the availability of genomic resources, its relevance in aquaculture, and its physiological sensitivity to environmental and nutritional factors. Establishing reliable methods for EV‐RNA isolation, storage, and analysis in this species will not only advance fundamental research on EV biology in fish but also enable applied studies on stress biomarkers and health monitoring in other farmed species.

The present study aimed to (i) evaluate the performance of the exoRNeasy kit for EV‐RNA isolation from fish plasma across different kit formats and input volumes; (ii) assess the impact of storage at ‐20°C versus fresh processing on RNA yield, composition, and transcript integrity; and (iii) provide a comprehensive molecular characterization of EV‐RNA cargo including mRNAs and lncRNAs using long‐read sequencing. By addressing both methodological and biological questions, this work contributes to establishing a framework for robust EV transcriptomics in aquatic vertebrates. This study is inherently exploratory, particularly in the context of long non‐coding RNAs, as no previous research has comprehensively characterized this RNA biotype in fish plasma EVs, underscoring the novelty and foundational nature of our work.

## 2 Materials and Methods

### 2.1 Sample Collection

*Sparus aurata* samples were collected from individuals reared at the Canary Islands Oceanographic Centre (COC, IEO‐CSIC) located at Santa Cruz de Tenerife, Spain. Specifically, whole blood from 13 individuals (mean weight: 1.08 ± 0.13 kg; mean length: 35.42 ± 1.48 cm) was collected for the purpose of this experiment. Morphometric and experimental data of each specimen are displayed in Table S1. These individuals were raised on black‐walled squared tanks of 10 m^3^ at a density of 1200 g/m^3^, an open water flow system with a flow of 80 L/min, a temperature range between 19.9‐20.4°C and dimmed natural photoperiod. Commercial diet (R‐10 Europe 22%, Skretting, Spain) was used as the main feeding source three times per week (on Mondays, Wednesdays, and Fridays) at a ratio of 2% of the tank biomass. Prior to blood collection, individuals were anaesthetized using MS‐222 (tricaine methanesulfonate) at 80 mg L^−1^. Whole‐blood collection was performed with 5 mL caliber 21 (21G) syringes and blood was collected in 1.5 mL Eppendorf tubes. Both syringes and tubes were treated with EDTA as anticoagulant instead of using Heparin due to potential interferences of the latter with wet lab protocols (QIAGEN 2019).

After sample collection, whole blood was centrifuged (2500 x g for 20 minutes at 4°C) and plasma was filtered to remove cellular debris using 0.8 µm Sartorius Minisart NML (Théry et al. 2018).

All experimental procedures were performed according to the EU Directive 2010/63/EU for animal experiments and the Spanish Executive Order 53/2013 for the protection of animals for experimentation or other scientific purposes. The experimental design was previously approved by the CSIC Ethical Committee and authorized by the National Competent Authority (CEIBA 1795/2024).

### 2.2 Exosomal RNA Isolation and Experimental Design

EV‐RNA was isolated from pre‐filtered plasma using exoRNeasy commercial kit (Qiagen, Hilden, Germany) following the manufacturer's instructions (QIAGEN 2019). This commercial kit is available in two different formats (Maxi and Midi) that differ in the amount of input material that can be processed (1000–4000 µL for the Maxi format, and 100–1000 µL for the Midi format) (QIAGEN 2019). For this article two different parameters were evaluated: (1) kit/volume format and (2) pre‐processing treatment. For the kit/volume comparison, plasma samples were compared immediately after collection (Fresh) using either Maxi (∼2 mL) or Midi format (∼1 mL). Due to volume limitations, several samples were combined to reach the necessary volume to be processed with the Maxi format. The pooling scheme for samples P1, P2, and P3 is shown in Table S1. For the pre‐processing treatment comparison, fresh plasma samples processed with the Midi kit (Fresh) were compared to those stored at ‐20°C for two weeks after collection and prior processing (Frozen). All the samples in this comparison were processed using the Midi kit to avoid having two parameters being compared simultaneously.

### 2.3 Quality Control

#### 2.3.1 Quality Control of Isolated EVs

For Quality control of the vesicles EV were eluted with EV elution buffer (Buffer XE, QIAGEN) prior to RNA isolation step. Quality control of isolated EVs was carried out by different complementary approaches. Dynamic Light Scattering (DLS) was used to assess EV size distribution through the particle and micro‐surface characterization service at Universidad de La Laguna facilities (SEGAI). DLS was performed on a Zetasizer Nano ZS (Malvern) equipped with a HeNe 633 nm laser and operating on a 173 nm angle. Measurements were performed at a fixed position, controlled temperature, and automatic attenuation. Transmission Electron Microscopy (TEM) was used to assess EV integrity, shape and size. TEM imaging was carried out with a JEM‐1400 Flash microscope through the Microscopy Service at Universidad de La Laguna (SEGAI). TEM was conducted on a single sample fixed using two different methods: freezing and 2.5% glutaraldehyde. This technique was used to confirm the presence, morphology, size, homogeneity and overall physical characteristics of the potentially isolated EVs.

#### 2.3.2 RNA Integrity and Quality

Following EV‐RNA isolation, RNA quality and integrity was assessed using a 4200 TapeStation system (Agilent) at the Genomics Service of the Technologic Institute of Renewable Energies (ITER). RNA integrity (RINe) values were obtained per assay. However, RINe is not very informative due to the characteristic enrichment in short RNAs, and low‐abundance of ribosomal RNAs (rRNAs) in EV‐RNA, contrary to tissue samples. Thus, an alternative index Short /Long ratio was used to assess RNA integrity. For that, the short fraction was defined as those RNAs transcripts with lengths between 35 and 200 bp to avoid overlapping with calibration markers, the long fraction as those transcripts longer than 200 bp, and the Short/Long ratio as the quotient between those two previously defined parameters.

### 2.4 RNA Quantification

RNA quantification was carried out using Qubit High Sensitivity Assay kit (Thermo Fisher Scientific) with fresh calibration standards for each assay. RNA yield was estimated per sample by multiplying the concentration by the elution volume. To account for differences in input volumes and kit format, yield was further normalized by the sample input volume, ensuring comparability across samples.

### 2.5 EV‐associated RNA Sequencing and Data Analysis

#### 2.5.1 Sequencing Libraries Preparation, Sequencing, and Basecalling

After RNA quality control, samples were pooled by pre‐processing treatment in two groups regardless of the kit format used to processed them (Fresh pool including samples M1, M2, and M3, and Frozen pool including samples MC1, M10, and M11) to ensure enough RNA input for Oxford Nanopore Technologies (ONT) sequencing. PCR‐cDNA barcoding kit 24 v14 (SQK‐PCB114‐24‐ONT), was used to prepare sequencing libraries assigning a different molecular barcode (BC) per pool (Fresh pool: BC3; Frozen pool: BC4) to enable the demultiplexing of the output reads with regards to the pre‐processing treatment. The sequencer used was a MinION Mk1C (ONT) property of the IEO. Sequencing data was processed with MinKNOW 24.11.8 and simultaneous base‐calling was performed using Dorado 7.6.7. with default parameters.

#### 2.5.2 Quality Control of Sequencing Data

Raw sequencing data were demultiplexed and quality‐filtered within MinKNOW suite using default settings. Subsequent sequence processing was performed using the wf‐transcriptomes workflow (v1.7.0) (https://github.com/epi2me‐labs/wf‐transcriptomes) through the open‐source platform EPI2ME (v5.2.5). This workflow was used to remove residual primers, adapters and barcodes, and perform the alignment against the reference genome (fSpaAur1.1) using the default parameters except the following: input format: fastq, reference‐guided option, direct_rna: false, pychopper_opts: ‐u unclassified_reads.fastq ‐l len_fail_reads.fastq ‐w rescue_reads.fastq ‐K qc_fail_reads.fastq, pychopper_backend: phmm, cdna_kit: SQK‐PCB114, minimap2_opts: ‐uf ‐y, minimum_mapping_quality: 30, stringtie_opts: –conservative.

*Sparus aurata* last version's reference genome (fSpaAur1.1), which includes 24 chromosomes and one organelle, was downloaded from the National Centre for Biotechnology Information (NCBI), and RefSeq annotation was selected for further analyses. Gene names as well as gene description were queried from the genomic annotation file (GTF) of the reference genome.

#### 2.5.3 Subsampling, and Transcript Quantification and Decay Analysis

To avoid a potential bias derived from differences in the number of reads obtained after sequencing between Fresh and Frozen pools, random subsampling was performed using the number of alignments of the pool with fewer alignments as the subsampling threshold. This random subsampling was performed three times using different seeds to discard stochasticity as the driven force of the results. BAM files per pool from wf‐transcriptomes output were converted to fastq using samtools v1.22 (Danecek et al. 2021), subsampling of fastq files was performed with seqtk software (v1.4‐r122) (Li n.d.), and read identifiers from the subsampled fastq files were used to filter the original BAM file of the pool with more alignments, generating three replicate BAM files that were used in downstream analyses. Transcript counting was performed in R (v4.4.2) using the *summarizeOverlaps()* function from the *GenomicAlignments* package (v1.42.0) with the following settings (*mode: IntersectionStrict, inter.feature: False, ignore.strand:True*) (Lawrence et al. 2013). Transcript counts were obtained independently for different RNA biotypes based on the information contained in the GTF file of the reference genome using only the gene entries (i.e., messenger RNA [*protein_coding*], long non‐coding RNA [*lncRNA*], small non‐coding RNA [*snoRNA and snRNA*], and other RNAs that do not fit in any of the previous categories [rRNA, tRNA, pseudogene, transcribed_pseudogene, C_region, V_segment, guide_RNA]). The ratios between the different RNA biotypes (lncRNA/mRNA, sncRNA/mRNA, and lncRNA/sncRNA) were evaluated as markers of transcript decay due to the pre‐processing treatment. Transcript decay of the transcripts annotated and detected in each sample was further assessed using an in‐house pipeline that executes FLAIR (v2.2.0) correct and collapse modules (Tang et al. 2020), followed by SQANTI3 (v5.5.1) quality control module (Pardo‐Palacios et al. 2024) with the default settings except ss_window parameter that was set to 90 in FLAIR's correct module.

#### 2.5.4 Transcript Functional Exploration and Enrichment Analyses

The most abundant transcripts in each RNA biotype category were queried on different databases to infer potential biological functions of the *in‐cargo* content of EVs. Functional enrichment analysis for mRNA and lncRNA categories independently (including all the transcripts in each category) was carried out with the R package *gprofiler2* (v0.2.3) (Kolberg et al. 2020, Kolberg et al. 2023) to assess if the transcripts detected in the EVs participate in specific functions or molecular compartments that could hint at the potential role they exert in EVs, and if any term was preferentially enriched in a specific pre‐processing treatment. Results were filtered based on *p*‐value, and only GO TERMS enriched in the Fresh pool with *p*‐value < 0.01 are reported in the Results section. To ease visualization and comparisons between Fresh and Frozen conditions parent–child term relationships were simplified, and redundant GO terms were clustered based on semantic similarity computed with the *GOSemSim* (v2.28.1) (10.18129/B9.bioc.GOSemSim) and *GO.db* (v3.18.0) (10.18129/B9.bioc.GO.db) packages (Lin method). Only the enriched GO terms with a p_adj_‐value < 0.01 were included. Hierarchical clustering (average linkage, similarity cutoff =  0.5) was applied to group related GO terms, and representative terms were selected as cluster centroids. This approach enabled the assessment of whether pre‐processing treatment biased the characterized transcript functions.

#### 2.5.5 Interaction Analyses Between lncRNAs, and mRNAs and Their Encoded Proteins

To elucidate the biological roles of the most abundant lncRNA transcripts in EVs, a pragmatic approach was adopted by predicting their interactions with mRNAs, and RNA‐binding proteins (RBPs) coded by the most abundant mRNAs in EVs, and analyzing the associated biological processes. Potential lncRNA–mRNA and lncRNA–RBP interactions were explored using Python (v3.10.19). Candidate mRNAs were identified through two sequential filtering steps with RNAplex (Lorenz et al., 2011) and IntaRNA (Mann et al. 2017), both executed via Python's subprocess module. The first filtering step aimed to rapidly select top mRNA candidates using RNAplex, a fast and efficient predictor of RNA–RNA interactions based on a simplified energy model that identifies likely base‐pairing regions without full RNA folding consideration (Tafer and Hofacker 2008). Only interactions showing an average energy per nucleotide < ‐0.03 kcal/mol/nt were retained as top candidates (Lai and Meyer 2016). The second filtering step refined this selection using IntaRNA, which predicts RNA–RNA interactions by integrating hybridization energy and site accessibility, penalizing regions that are structurally constrained (Mann et al. 2017). Interactions with an average energy per nucleotide < ‐0.3 kcal/mol/nt were kept as final candidates to ensure a more selective and biologically relevant subset.

Interaction analysis between lncRNA and RBP was performed in three steps. First, among the most abundant mRNAs, genes likely coding for RBPs were identified based on biological annotations (NCBI descriptions). Next, the corresponding protein sequences of these mRNAs likely coding for RBPs, were retrieved to predict their potential interactions with the most abundant lncRNAs. This was achieved using a machine learning RNA‐protein predictive pipeline, inspired by the conceptual framework of IPMiner (Pan et al. 2016). The model was trained on several widely used RNA‐protein interaction (RPI) benchmark databases including *RPI1807*, *RPI369*, *RPI13254*, *RPI488*, and *NPInter10412*, which cover diverse RNA biotypes (e.g., lncRNAs, mRNAs) and protein sequences. Proteins were encoded via the Conjoint Triad method (Shen et al. 2007) using 7 amino acid groups and 3‐mer combinations, which led to a first pool of 343 (7^3^) features. RNAs were encoded via nucleotide 3‐mer frequencies (4 nucleotides leading to 4^3^ = 64 features). A total of 407 concatenated features were compressed into a 64‐dimensional latent space using a stacked autoencoder, which captures non‐linear relationships between protein and RNA features and reduces noise. The encoder network was saved and reused to transform new feature vectors during prediction. Then, a Random Forest classifier was trained on the encoded features and validated using 5‐fold stratified cross‐validation to ensure balanced evaluation across interaction classes. The decision threshold for strong interactions was chosen preferentially to maintain a low false positive rate (≤ 0.10). Predicted interactions were reported as probabilities, and top‐scoring protein–lncRNA pairs were saved. This pipeline enabled rapid and high‐confidence prediction of lncRNA–protein interactions with interpretable feature representation.

A lncRNA–mRNA interaction network was built for the most abundant lncRNAs found in all the samples. Since GO terms were not available for many mRNAs, manual functional annotation was performed based on predicted gene identity, and only manual annotated functions with ≥ 5 genes were included in the network. Nodes represent the lncRNA or its associated mRNAs, which were grouped by functional categories. Edges connect the lncRNA to each mRNA, and node size encodes interaction energy scores. A force‐directed layout (Fruchterman–Reingold algorithm) was used with manual adjustments to improve cluster separation.

### 2.6 Statistical Analyses

All statistical analyses were carried out in R (v4.4.2). Normality of quantitative variables (RNA integrity and quantification metrics) was tested using the Shapiro‐Wilk test (shapiro.test()) in each of the three test groups (Fresh‐Maxi, Fresh‐Midi and Frozen‐Midi) (J. P. Royston 1982b, 1982a; P. Royston 1995). Homoscedasticity was primarily evaluated using Levene's test (leveneTest() from the *car* package (v3.1.3) (Fox et al. 2001), which is robust to non‐normal distributions. Due to small sample size, for completeness, F‐tests (var.test()) and Bartlett's test (bartlett.test()), both from the *stats* package (v4.4.2), were also performed.

If normally distributed, and homoscedastic, a t‐test for independent samples was performed to assess differences between groups. Given the small sample size (n =  3), Wilcoxon rank‐sum test (wilcox.test()) from the *stats* package (v4.4.2) was also performed as a non‐parametric alternative. Additionally, permutational analyses (oneway_test()) from the *coin* package (v1.4.3) were also performed to assess for differences between groups (Hothorn et al. 2006, Hothorn et al. 2008), and non‐parametric bootstrap analyses were carried out to estimate robust confidence intervals (Efron and Tibshirani 1994).

For sequencing data, the Kolmogorov‐Smirnov test from the *stats* package (v4.4.2) was applied to evaluate potential differences in the transcript length distributions between Fresh and Frozen samples (considering both the original pools as well as the subsamples). Chi‐square test was used to evaluate potential differences between transcript lengths within bins of preset sizes (100, 250, and 500 bp) in order to identify transcript length bins where samples differ in terms of absolute abundance, which could be used as a proxy of RNA degradation.

## 3 Results

### 3.1 EV Quality Control

The average diameter (Z‐average) was 418.1 nm with a main peak at 362.8 nm, as shown by the DLS report, which suggests a unique population of EVs with a medium‐to‐large size (Figure S1). A polydispersity index (PDI) of 0.428 pointed to a slightly heterogeneous distribution. A standard deviation of 68.50 nm suggests that most of the EVs have a diameter around the main peak.

Morphological characterization of the EVs was performed on two aliquots of a randomly selected sample using TEM. Micrographs show EV‐compatible structures with spheric to oval shape, clear edges, and a relatively clear luminal space (Figure S2), consistent with previous literature for this type of biological sample (Kowal et al. 2014; Théry et al. 2018). Panels A‐D of Figure S2 correspond to EVs from the aliquot fixed with Glutaraldehyde. These micrographs show structures with a typical EV appearance, homogeneous size distribution and a diameter smaller than 200 nm. In particular, panels A and B predominantly show independent spherical structures, while panels C and D show greater size heterogeneity and a higher density, resulting in small complexes. Panel E shows the cryo‐fixed aliquot, exhibiting lower contrast and a less‐defined morphology with blurred edges in comparison with the glutaraldehyde‐fixed fraction. The granular appearance observed might be due to a chemical interaction between the contrast agent and the EV elution buffer (Buffer XE, QIAGEN) which could produce a pellet or alter the distribution of the contrast agent on the grid, potentially affecting the definition of the images (Cheville and Stasko 2014).

### 3.2 RNA Integrity and Length Distribution

Electrophoretic profiles showed low intensity 18S and 28S rRNA peaks for all samples (Figure S3A–I), along with a high proportion of short length transcripts, with two peaks at ∼25 and ∼75 bp (Figure S3A–I). This was the pattern expected from EV‐RNAs which usually are enriched in short RNAs such as microRNAs (miRNAs) or transference RNAs (tRNAs) compared to tissue samples (Pelyhe and Sturve 2023; Solaguren‐Beascoa et al. 2023; Torres and Martí 2021). The Short/Long ratio had values over unity in all of the samples, confirming the higher abundance of short transcripts over long transcripts (Table S1), while RNA size distribution indicated minimal degradation, supporting the efficiency of the EV isolation protocol (Enderle et al. 2015; Granada et al. 2018). The peak of ∼25 bp was excluded from these estimations despite potentially representing miRNAs due to an overlap with the calibration marker, while peak between 35–90 bp (maximum at 75 bp) reflected an enrichment in short transcripts compatible with tRNAs (as full transcripts or partially degraded). Due to the low rRNA signal, the 28S/18S ratio may be inconsistent across samples and is likely uninformative for evaluating EV‐RNA quality. Therefore, it should not be relied upon for group comparisons.

### 3.3 Kit Format Comparison (Maxi Format vs. Midi Format)

#### 3.3.1 RNA Integrity and Length Distribution

Statistical analysis of the 28S/ 18S indicated slightly higher ratios in Maxi compared with Midi samples (*p*‐value = 0.1) (Figure S4A and Table S5).

The Short/Long index did not differ significantly between kit formats (*p*‐value > 0.05) (Figure S4C and Table S5).

These results suggest that kit format choice does not affect the quality of RNA isolated from EVs.

RNA concentration, yield, and normalized yield

Concentration of the eluted RNA as well as yield, and input‐volume normalized yield were compared between the different kit formats (Table S1), and all three quantification variables were normally distributed and homoscedastic (Table S5).

No differences were found between Maxi and Midi formats for any of the variables considered (*p*‐value > 0.05) (Figures 1A and S4E,G,I and Table S5). These results suggest that kit format choice does not affect the efficiency of the RNA isolation from EVs.

**FIGURE 1:**
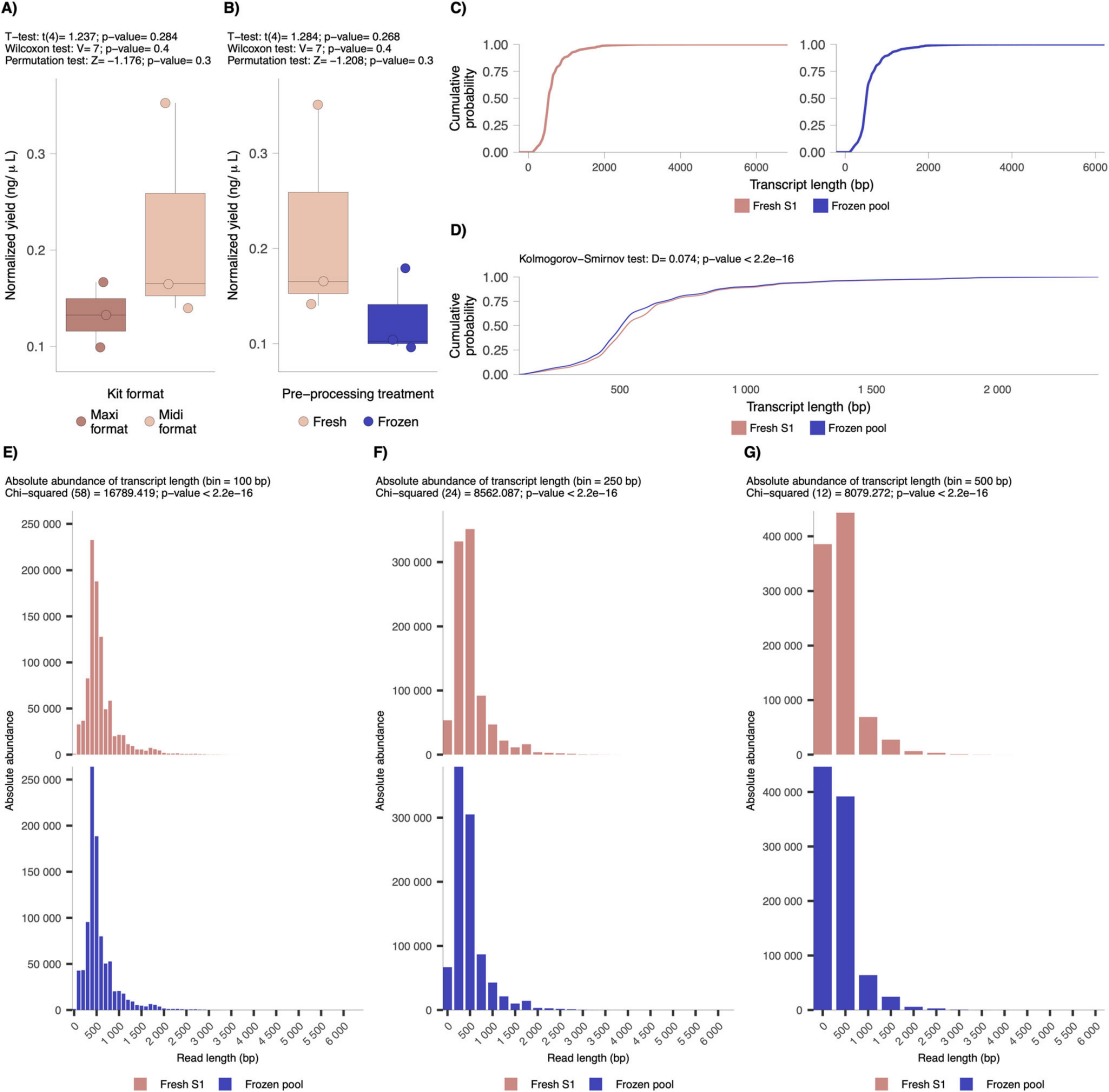
Normalized yield and transcript length distribution results. (A) Boxplot of normalized yield (ng/µL) comparing kit formats: Midi (∼1 mL) vs. Maxi (∼2 mL); (B) Boxplot of normalized yield (ng/µL) comparing pre‐processing treatments: Fresh vs. Frozen (−20°C); (C–G) Transcript length distribution comparisons for Fresh subsample 1 (Fresh S1) vs. Frozen pool [Fresh sample selected as representative; for full analysis, see Figure S5, comparing Fresh subsamples and Frozen pool transcript length distributions across different bin sizes]; (C–D) Cumulative distribution of transcript lengths: (C) Full cumulative distribution for each sample; (D) zoomed‐in view highlighting differences among transcript length distributions;(E–G) Histograms of transcript length distributions using bin sizes of 100 bp (E), 250 bp (F), and 500 bp (G).

### 3.4 Pre‐processing Treatment Comparison (Fresh vs. Frozen Samples)

#### 3.4.1 RNA Integrity and Length Distribution

Statistical analysis of the 28S/18S showed no significant differences between pre‐processing treatments (Fresh vs. Frozen) (*p*‐value > 0.05) (Figure S4B and Table S5).

The Short/Long ratio did not differ significantly between pre‐processing treatments (*p*‐value > 0.05) (Figure S4D and Table S5).

All in all, these results suggest that pre‐processing treatment does not affect efficiency of RNA isolation from EVs.

#### 3.4.2 RNA Concentration, Yield, and Normalized Yield

Concentration of the eluted RNA as well as yield, and input‐volume normalized yield were compared between the different pre‐processing treatments (Table S1), and all three quantification variables were normally distributed and homoscedastic (Table S5).

For the pre‐processing treatment comparison, no significant differences were found for any of the variables (*p*‐value > 0.05) (Figures 1B and S4F, H, and J and Table S5). However, frozen samples yielded slightly lower RNA concentrations and total RNA amounts, even after normalization by input volume, compared to fresh samples (marginal trend; *p* = 0.1). Given the constraint of a limited sample size, the bootstrap resampling approach was employed to approximate the empirical distribution of mean differences across groups, thereby enabling the estimation of a robust confidence interval. This non‐parametric approach could provide additional validation for the previously observed trends for the RNA yield between fresh and Frozen samples. Estimated confidence interval (0.080 – 0.194) suggests differences in RNA yield between groups, however those differences were not statistically significant (*p*‐value _bootstrap_ = 0.498). These results suggest that storing blood plasma samples at ‐20°C for two weeks does not have a substantial effect on RNA isolation efficiency, although a subtle impact cannot be ruled out.

#### 3.4.3 RNA Sequencing

After sequencing, Fresh and Frozen pools retrieved 3,442,247 and 1,084,806 quality‐filtered reads, respectively. After bioinformatically removal of barcode sequences, sequencing adapters, and primers, the Fresh pool retained 2,980,654 aligned reads (86.59%) while the Frozen pool retained 936,415 aligned reads (86.32%). To allow a fair comparison, Fresh BAM file was subsampled to 936,415 primary alignments, matching the Frozen pool, and eliminating read count as a potential confounder biasing between‐groups comparison.

Regarding the length distribution, the mean length of reads was 634.5 ± 375.6 for the Fresh pool (634.5 ± 376.3 for the Fresh subsample 1 (Fresh S1), 634.8 ± 376.3 for the Fresh subsample 2 (Fresh S2), 634.7 ± 376.0 for the Fresh subsample 3 (Fresh S3)) and 606.5 ± 368.9 for the Frozen pool. An enrichment in transcripts with lengths under ∼500 bp was observed in all samples regardless of their pre‐processing treatment or subsampling status. Due to the protocol followed for the sequencing‐library preparation, transcripts shorter than ∼200 bp were not sequenced, despite being present in the samples, as depicted in the TapeStation quality plots (Figure S3).

Length distribution among the Fresh subsamples was compared using a two‐sample Kolmogorov‐Smirnov test, and no differences were found (Fresh S1 vs Fresh S2: D = 0.001, *p*‐value = 0.710; Fresh S1 vs Fresh S3: D = 0.001, *p*‐value = 0.984; Fresh S2 vs Fresh S3: D = 0.001; *p*‐value = 0.949), indicating similar distributions. In contrast, when comparing Fresh samples against the Frozen pool, all the comparisons revealed statistically significant differences (*p*‐value < 0.001) (Figure S5). Cumulative distribution plots showed that the Frozen pool had a higher number of shorter reads in comparison with Fresh samples (Figures 1D and S5). Chi square test further confirmed statistically significant differences between length distributions at 100‐pb bins, 250‐bp bins, and 500‐bp bins (*p*‐value < 0.001) (Figures 1E–G, S5 and S6). The distributions of Chi‐square standardized residuals revealed that the intervals that contributed the most to the observed differences were: the overrepresentation of shorter reads (100–500 bp) in the Frozen pool, and longer reads (600–2400 bp) in the Fresh samples (Figures S5 and S6).

#### 3.4.4 Genomic Characterization of EV Transcripts

Reference genome of *Sparus aurata* contains 32,008 gene entries according to the annotation file in Gene transfer format (GTF) from which 25,735 correspond to protein‐coding genes, 3,538 to lncRNAs, 231 to snoRNAs, 159 to snRNAs, 1,268 to tRNAs, 96 to rRNAs, 601 to pseudogenes, 17 to transcribed pseudogenes, 297 to immunoglobulin variable segment, 58 to immunoglobulin constant region, and 8 to guide RNAs (Table S2).

Transcripts were annotated to 7,873.4 ± 1,803.73 protein‐coding genes, 223.2 ± 57.40 lncRNA genes, 78 ± 22.55 other RNAs, and 8.8 ± 1.92 sncRNA genes, with Frozen pool consistently showing the lowest gene representation (Table S2). In terms of transcript count, mRNAs represent the predominant RNA biotype (1,183,513.4 ± 804,370.63) in both the Frozen pool and Fresh subsamples, followed by lncRNAs (66,807.4 ± 44,744.57), other RNAs (2,282.6 ± 1,661.04), and sncRNAs (42.4 ± 27.53) (Figure 2A and Table S2). The Fresh pool followed the same distribution pattern but contained higher read numbers overall (mRNA =  2,622,415, lncRNA =  146,804, other RNAs =  5,232, sncRNA =  73), reflecting the greater sequencing depth obtained for this sample. While the Frozen pool resembled Fresh subsamples in read distribution across most RNA biotypes, it diverged for sncRNAs (Figure 2A and Table S2).

**FIGURE 2:**
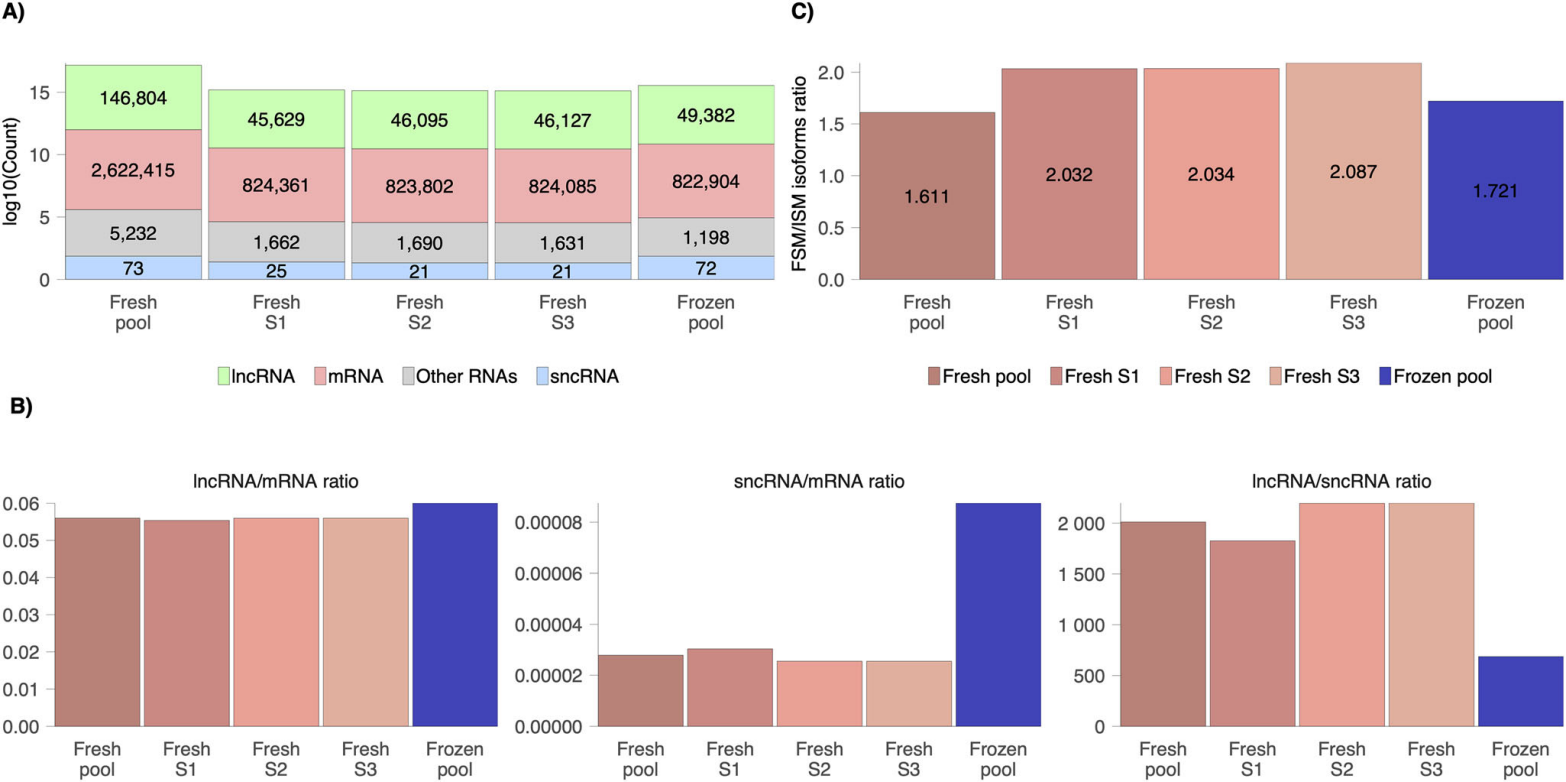
Impact of plasma pre‐processing (freezing) on RNA biotype composition and transcript completeness. (A), Stacked bar plot showing the abundance (log_10_ counts) of each RNA biotype; (B) Ratios between major RNA biotypes: lncRNA/mRNA, sncRNA/mRNA, and lncRNA/sncRNA;(C) Ratio of full‐length versus incomplete isoforms (FSM/ISM).

To explore a potential selective enrichment or depletion of RNA biotypes associated with freezing, the lncRNA/mRNA, sncRNA/mRNA, and lncRNA/sncRNA ratios were assessed. Ratios between the main transcript categories revealed notable differences in the relative distribution of reads annotated to each RNA category. The lncRNA/mRNA ratio was similar between Fresh and Frozen samples. On the other hand, sncRNA/mRNA ratio was higher in the Frozen pool (Fresh pool =  2.78×10^−5^; Fresh subsamples range S1‐S3 = 2.55×10^−5^ ‐ 3.03×10^−5^; vs Frozen pool =  8.75×10^−5^), and lncRNA/sncRNA ratio was higher in the Fresh samples (Fresh pool =  2011.01; Fresh subsamples range S1‐S3 =  1825.16 ‐ 2196.52 vs Frozen pool =  685.86). These observations hint to a relative increase in reads annotated to sncRNAs in the Frozen pool compared to the Fresh samples (Figure 2B and Table S2).

Reads assigned to mRNA gene entries covered 10,230 protein‐coding genes of which the 50 most abundant represented over a 60% of all the reads in each sample (Fresh pool and subsamples S1‐S3 =  0.64; Frozen pool = 0.62). Nearly all the top 50 genes (∼95%) were consistently in the top 50 in both Fresh and Frozen samples. The vast majority (85%) of these top 50 protein‐coding genes included ribosomal proteins or ribosome‐associated proteins (Table S3). When comparing the transcript ID for those with a relative abundance above 0.01% (>0.0001) accounting for over 90% of the reads in Fresh subsamples and the Frozen pool, 374 out of 376 transcripts shared in the Fresh subsamples, were also detected in the Frozen sample (393), with again over 95% of the genes found in both Fresh and Frozen samples.

Reads assigned to lncRNA gene entries mapped 304 lncRNA genes none of which is currently characterized. Of the 304 lncRNA genes, two accounted for almost 90% of the reads regardless of the sample (Fresh pool =  88%; Fresh S1 =  88%; Fresh S2 =  88%; Fresh S3 =  88%; Frozen pool =  90%) (Table S3).

#### 3.4.5 Isoform Identification Analysis

Analysis of SQANTI3 output revealed that the Fresh pool retrieved the highest number of isoforms (11,738), followed by the three Fresh subsamples (Fresh S1 = 6,685, Fresh S2 =  6,624, and Fresh S3 = 6,611) and the Frozen pool (5,252) (Table S6). Across all samples, full‐splice match isoforms (FSM) were the most abundant isoform type, followed by incomplete‐splice match isoforms (ISM). The ratio between FSM vs. ISM (FSM/ISM) was generally higher in Fresh samples, which indicates a higher proportion of incomplete isoforms detected in the Frozen pool (Fresh pool = 1.61; Fresh S1 =  2.03; Fresh S2 = 2.03; Fresh S3 = 2.09; Frozen pool = 1.72) which could reflect RNA degradation during freezing or storage (Figure 2C).

### 3.5 Enrichment Analysis

Enrichment analyses using the *gprofiler2* R package were performed for mRNAs and lncRNAs detected in the EVs, analyzed independently sample and RNA biotype‐wise (Table S4).

For the mRNAs, 148 terms related to Biological processes (BP), 81 terms related to Cellular component (CC), and 60 terms related to Molecular function (MF) and 140 terms related to Human Phenotype Ontology (HP) were selected. In general terms, the enrichment profile was similar across samples, although some terms were specific to Fresh or Frozen samples. An enrichment in BP terms related with translation, ribosome biogenesis, rRNA processing, intracellular transport, protein transport, endosomal transport, autophagy, protein maturation, establishment of localization in cell, regulation of organelle organization, RNA localization, hemopoiesis, protein folding was observed. CC enriched terms included intracellular organelle, intracellular membrane‐bound organelle, nucleus, intracellular vesicle, endosome, Golgi apparatus, cytosolic ribosome, RNA polymerase complex, organelle subcomplex, vesicle, or early endosome. Lastly MF terms were related to binding to different molecules such as ribonucleotides, ATP, ions or modification of histone activity or rRNA binding (Table S4). Top‐enriched GO terms include translation, protein metabolic processes, intracellular transport, vesicle‐mediated transport, and autophagy (Figure 3A). Overall GO BP terms enrichment was similar for Fresh and Frozen conditions (Figure 3A,B and Table S7).

**FIGURE 3:**
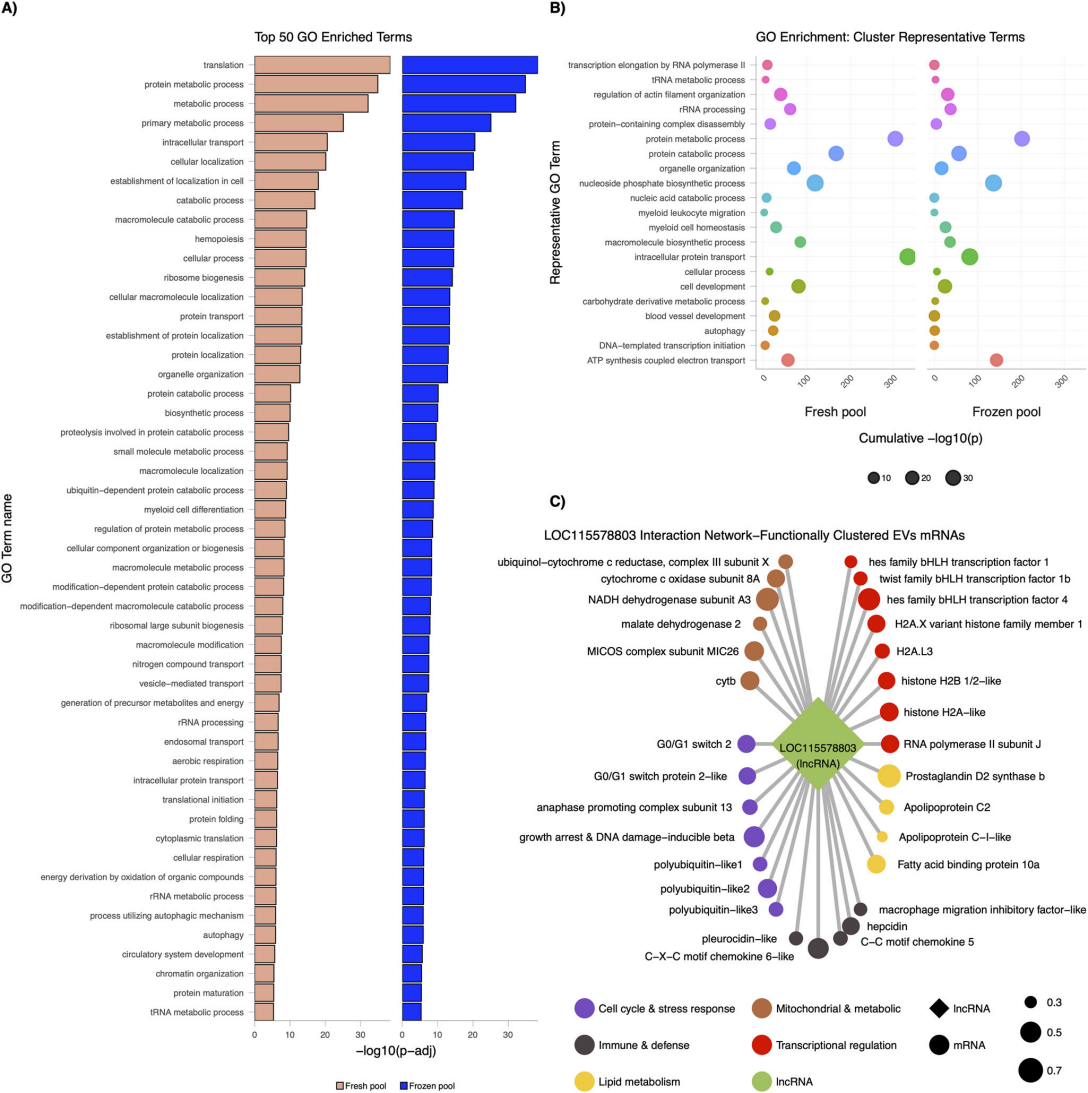
Summary of the mRNA GO: TERM enrichment and lncRNA‐mRNA interaction analyses. (A) Top 50 GO terms enriched in Fresh and Frozen samples. (B) bubble plot representing clustered semantically similar GO terms. To reduce redundancy and ease visualization parent–child term relationships were simplified, and redundant GO terms were clustered based on semantic similarity. The x‐axis represents the cumulative *p*‐value calculated as the sum of ‐log_10_(*p*‐value _adj_) of all the terms included in each cluster, reflecting the overall enrichment significance, and the y‐axis represent each of the clusters, with the size of each dot representing the number of GO terms included in each cluster, while color encodes the representative GO term; (C) lncRNA–mRNA interaction network generated for *LOC115578803*. Nodes represent the different transcripts assessed, with different node shapes representing transcript biotype, node size representing the absolute value of interaction energy (|Ie|) between the *LOC115578803* and the different mRNAs, and colors representing the different manually annotated functions among the mRNAs that interact with *LOC115578803*. Only functional groups containing five or more genes were included in the network.

No enrichment was found when considering lncRNAs since no functional annotation was available for any of the genes queried. Therefore, to explore their potential functions within EVs, interactions between the two most abundant lncRNAs (*LOC115584152* and *LOC115578803*) and the pool of mRNA transcripts contained in the EVs were investigated.

*LOC115584152* was predicted to interact with 16 mRNAs from the ones detected in the EVs (Table S8), including several genes involved in mitochondrial function and oxidative phosphorylation (3), transcriptional regulation and chromatin dynamics (3), signaling pathways (2), oxygen transport, sensory processes, and immune defense.

*LOC115578803* was predicted to interact with 94 mRNAs (Figure 3C and Table S9). The 94 predicted target mRNAs include transcription factors, mitochondrial and ribosomal components, immune‐related genes, metabolic enzymes, and structural proteins (Figure 3C and Table S9). Interestingly, 12 out of the 94 mRNA that potentially interact with *LOC115578803*, also interact with *LOC115584152* (Tables S8 and S9).

Given that lncRNAs often exert their functions through interactions with RNA‐binding proteins (RBPs), we further explored the potential associations between the two most abundant EV‐associated lncRNAs (*LOC115584152* and *LOC115578803*) and the ribonucleoproteins (RNPs) encoded by the most highly represented EV‐mRNAs. Remarkably, 40 of the proteins encoded by these mRNAs were predicted to interact with one or both lncRNAs (Tables S10 and S11).

## 4 Discussion

EV‐RNA has emerged as a promising biomarker source in aquatic models (Moros et al. 2021; Zhao, Deng et al. 2021). However, pre‐analytical variables, such as plasma volume and storage conditions, could significantly influence RNA yield and cargo composition, particularly in low‐input samples like fish plasma. In this study, we assessed the impact of plasma volume and freezing on the quantity and quality of RNA purified from EVs isolated from *Sparus aurata* plasma. Using a standardized isolation workflow combined with cDNA‐PCR barcoding for Oxford Nanopore Technologies sequencing, we compared RNA yield, integrity, transcript size distribution, biotype ratios and relative proportions, as well as the abundance of full‐length versus incomplete isoforms between fresh and frozen plasma samples. In addition, we analyzed the transcriptomic profiles of the long RNA fraction, with particular emphasis on lncRNAs, as this represents a central element of our ongoing research hypothesis, which lies beyond the scope of the present study.

Overall, our results provide novel insights into how pre‐analytical handling impacts EV‐RNA recovery and transcriptomic representation in fish plasma. These findings offer an essential framework for optimizing EV‐based RNA workflows in aquatic species and for interpreting transcriptomic variability in downstream analyses. Although EV isolation and characterization from fish biofluids have been reported (Nikapitiya et al. 2022; Zhao, Deng et al. 2021), and the effects of storage on EV integrity and RNA have been investigated in mammalian systems (Ahmadian et al. 2024; Sivanantham and Jin 2022; Weber et al. 2024), there is a noticeable lack of studies that directly assess how plasma input volume and freezing influence EV transcriptomic profiles in fish, particularly using long‐read sequencing approaches. Consequently, to our knowledge this work is the first to directly evaluate these specific pre‐analytical variables on EV‐RNA transcriptomes in a teleost plasma model, providing an important methodological framework for future EV‐RNA biomarker studies in aquatic species. Furthermore, while extensive human plasma/serum EV‐long RNA literature demonstrates feasible detection and diagnostic potential EV‐lncRNAs, our study establishes a methodological framework for investigating EV‐lncRNAs in teleosts. In the following sections we interpret our findings with respect to methodological standardization, EV biology, and the implications for biomarker discovery in fish models.

EV characterization/quality control: Morphological and quality control analyses confirmed the successful isolation of vesicles consistent with the expected size, shape and RNA profiles of EVs. The plasma‐derived EVs obtained in this study showed a main peak at 362.8 ± 69.77 nm suggesting a predominant population of medium‐to‐large EVs. These values fall within the expected range for plasma EVs, where a mixture of microvesicles (50‐1000 nm) and exosomes (50‐150 nm) coexists (Pelyhe and Sturve 2023; Théry et al. 2018). The absence of relevant secondary peaks suggests minimal presence of macromolecular aggregates or contaminants, supporting the efficiency of the blood plasma collection and filtering, and the suitability of exoRNeasy kit as a valid alternative to other conventional isolation methods (An et al. 2018). The moderate polydispersity index (PDI = 0.43) indicates a heterogeneous yet biologically consistent size distribution (Ouahabi et al. 2021; Sanaee et al. 2023). This heterogeneity might reflect the diverse origin of the EVs or their dynamic nature (Baj‐Krzyworzeka et al. 2007; Szatanek et al. 2017). Transmission electron microscopy confirmed spherical morphology and overall uniform distribution, with vesicle diameters mostly below 200 nm and occasional mild clustering. While occasional mild clustering was observed, the vesicles retained structural integrity suggesting high‐quality isolation. The apparent discrepancy between the DLS peak (∼363 nm) and TEM sizes likely reflects the DLS sensitivity to larger particles and aggregates, emphasizing the complementary value of both techniques (Filippov et al. 2023). Overall, the TEM observations support that the isolated EVs are representative of plasma EV populations reported in the literature.

Electrophoretic RNA profiles showed faint 28S and 18S rRNA bands (<5%) and a high abundance of small RNAs, with two distinct peaks at approximately 25 and 75 nt. This pattern is typical of EV‐RNA, with a strong depletion of intact rRNAs compared to total cellular RNA, and enrichment in short RNA species such as miRNAs and tRNA fragments (Solaguren‐Beascoa et al. 2023; J. Wang et al. 2023). The Short/Long RNA ratio was consistently greater than 1, confirming the predominance of small RNAs (<200 nt) characteristic of EV cargo. The secondary peak between 35–90 nt, with a maximum around 75 nt, indicates enrichment in tRNAs or tRNA‐derived fragments (Pan et al. 2025).

Collectively, these morphological and molecular features validate the isolation of bona fide EVs from *Sparus aurata* plasma and establish a solid quality‐control framework for subsequent transcriptomic analyses.

### 4.1 Validation of the exoRNeasy Kit in *Sparus aurata*

The exoRNeasy kit efficiently isolated EVs and purified total RNA, including lncRNAs, from *Sparus aurata* plasma, establishing a reproducible protocol for a non‐model aquaculture species and representing the first validation of this kit in fish species.

Column‐based membrane‐affinity approaches such as exoRNeasy have been shown to provide efficient and reproducible recovery of vesicles and RNA from low‐volume plasma samples, often yielding equal or higher RNA purity and quantity compared to classical ultracentrifugation‐based methods (QIAGEN 2019), features that are particularly advantageous for low‐input fish plasma. Although EV isolation and RNA profiling have been described previously in teleosts (e.g., rock bream (Nikapitiya et al. 2022) and Chinese tongue sole (Zhao et al. 2020; Zhao, Zhang et al. 2021)), there are few to none published validations of the exoRNeasy workflow in marine teleost plasma. Previous studies using either ExoQuick‐TC (System Biosciences) or ExoEasy Kit (Qiagen) are effective for exosome isolation, but additional steps are required for RNA purification, increasing the risk of contamination or sample degradation. In contrast, exoRNeasy allows simultaneous vesicle capture and RNA purification, optimizing RNA yield and integrity for downstream applications such as RNA sequencing or qPCR.

Besides, EV content analyses in teleost fish, have mainly focused on small RNAs, including miRNAs and piRNAs (Magnadóttir et al. 2020; Pan et al. 2025; Smith et al. 2020; Zhao et al. 2020; Zhao, Zhang et al. 2021) and protein cargo (Zhao, Deng et al. 2021), while long‐chain RNAs, such as mRNA and lncRNAs, remain largely unexplored (Zhao, Deng et al. 2021). Here, we use long‐read (Oxford Nanopore Technologies) library preparation to standardize a protocol for profiling the long RNA fraction from EVs cargo. Although ONT sequencing is well suited for capturing long and full‐length RNA molecules, it is not appropriate for very short RNAs such as miRNAs or piRNAs. This limitation arises because ONT sequencing requires RNA fragments of sufficient length to maintain stable translocation through the nanopore; consequently, short RNAs (< 200 nt) are often inefficiently captured or lost during adapter ligation and library preparation (Soneson et al. 2019; Workman et al. 2019). For studies aiming to capture the full RNA profile, alternative sequencing platform and protocols or adaptations of the current protocol may be considered. For example, modifying library preparation to retain shorter or non‐polyadenylated RNAs—through adjustments in magnetic bead clean‐up ratios, enzymatic polyadenylation (ONT kits), or random‐primed cDNA synthesis—could broaden RNA species representation, though very short RNAs will remain underrepresented or entirely not captured.

Consequently, to the best of our knowledge, these results provide the first explicit demonstration that the exoRNeasy protocol yields RNA from *Sparus aurata* plasma EVs with the expected small‐RNA–enriched profiles (as confirmed by electropherograms), and is compatible with long‐read sequencing for long RNA EVs cargo analysis. To mitigate known limitations of affinity‐based capture, we combined exoRNeasy with orthogonal QC (TEM, size distribution, and RNA size distribution), which together support the vesicular origin of the recovered RNA and the suitability of this approach for EV‐RNA biomarker studies in teleosts.

### 4.2 Sample Volume and Yield

Comparative analysis of the exoRNeasy kit formats revealed no significant differences in RNA concentration, yield and normalized yield between the Maxi and Midi formats, which imply that both formats are equally efficient in isolating RNA from EVs at the volumes tested, as reported by manufacturer (QIAGEN 2019). Due to volume limitations, Maxi kit format was applied to pooled biological material to comply with format kit volume requirements, whereas Midi kit format was applied to individual samples. This comparison does not represent a controlled assessment of input volume alone. Therefore, the results should be interpreted as indicating comparable performance of the two formats in our experimental setting rather than demonstrating equivalence attributable solely to sample volume. Nevertheless, using different samples in this comparison ultimately reinforces the reproducibility of the extraction kit, given that, regardless of the biological background, no statistical variation was observed in terms of RNA concentration, yield or input volume‐normalized yield.

Although most comparative studies have been performed in mammalian plasma, they indicate that RNA yield and transcript diversity from EVs can depend on the starting plasma volume (Enderle et al. 2015), with even small volumes providing sufficient material for downstream analyses (Kong et al. 2023; Robinson et al. 2024). In teleosts, data are limited. Some work shows EVs can be recovered and analyzed from small volumes of fish plasma or cell culture (Nikapitiya et al. 2022; Smith et al. 2020), but systematic comparisons of volumes or methods are lacking, supporting the need for standardized input volumes in low‐volume samples. Our findings are particularly advantageous in aquaculture research, where biological samples such as fish plasma are often limited, and a single sample may need to be allocated across multiple assays to obtain multi‐layered molecular information. The ability to use different kit formats without compromising RNA recovery provides flexibility in experimental design and sample management, allowing flexibility in sample volume without compromising efficiency. Nevertheless, standardizing input volumes may still be advisable, since variations in sample volume beyond the tested range and biological background could influence column performance and/or extraction efficiency.

### 4.3 Impact of Preprocessing: Frozen vs Fresh

RNA integrity and quality observations from previous studies do not reach an agreement on the freezing‐derived effect. A systematic review of the effect of different storage and freezing protocols on EVs indicated that rapid freezing procedures and constant subzero temperatures (optimally −80°C) preserve EV quantity and RNA cargo effectively (Ahmadian et al. 2024). Nonetheless, Gelibter et al. (2022) and Tessier et al. (2021) showed a reduction in RNA after storing samples at ‐80°C, particularly in low biomass samples. Assessment of pre‐analytical variables in urinary samples also shows that −20°C causes measurable losses (Barreiro et al. 2021). In our study, although no statistically significant differences were detected, freezing tended to reduce RNA normalized concentration. Bootstrap analysis revealed a numerical trend toward lower RNA yield in frozen samples compared to fresh, but the confidence interval included values close to zero, indicating that any effect on overall RNA yield is likely small, suggesting that any true effect of storage on RNA yield is likely small and biologically subtle. This is consistent with previous reports showing that cryopreservation can lead to minor losses of EVs and their RNA cargo (Ahmadian et al. 2024; Tessier et al. 2021). Thus, our findings suggest that storing blood plasma samples at ‐20°C for two weeks does not have a substantial effect of RNA isolation efficiency, though a subtle impact cannot be entirely ruled out. This consideration is particularly important for low input samples with RNA concentration in the picogram range, where low‐abundant transcripts might be compromised by smaller RNA losses during isolation. Those could be accentuated by technical errors inherent to techniques such as qPCR or RNA sequencing. On the other hand, Enderle et al. (2015) indicate that high‐quality EV‐RNA yields can be obtained using affinity‐based methods at levels similar to or better than optimized ultracentrifugation, thus RNA yield might be less sensitive to freezing losses depending on matrix and isolation method of selection. Thus, the method selection for RNA isolation and purification from EVs can be crucial to the effect of sample processing.

Interestingly, in this study, freezing significantly affected transcript size distribution and isoform representation. Results were consistent regardless of interval size, indicating robust transcript size distribution differences. Furthermore, Fresh samples yielded more isoforms overall, suggesting better RNA integrity or more efficient library preparation compared to the Frozen pool. Across all samples, Full‐Splice Matches (FSM) were more abundant, indicating that most transcripts were captured completely. Incomplete Splice Matches (ISM), representing partially recovered transcripts, were relatively more abundant in frozen samples, suggesting a higher proportion of incomplete isoforms potentially due to degradation during freezing or storage. These findings support that freezing can induce non‐uniform degradation, leading to a higher proportion of shorter RNA fragments and a 3′ bias in transcript coverage which can alter transcript size distribution and reduce the accuracy of quantification, especially for longer transcripts or those with low abundance, which is consistent with prior studies assessing the broad impact of RNA degradation on RNA sequencing (Kellman et al. 2021; Prawer et al. 2023).

Freezing also appeared to affect RNA biotype representation. The higher sncRNA/mRNA ratio and lower lncRNA/sncRNA ratio in the frozen samples indicate a relative enrichment of sncRNAs, which could reflect their intrinsic structural stability (Childs‐Disney et al. 2022; Louvat et al. 2024) or protection within RNA–protein complexes (Bandyra et al. 2016; Yan et al. 2024), which can inherently protect them from exonucleases and other degradative enzymes. Conversely, the apparent decrease in mRNA representation may result from greater susceptibility of long, unstructured transcripts to degradation (Basu et al. 2021).

Despite previous reports indicating that freeze–thaw cycles and suboptimal storage conditions reduce EV numbers, increase particle aggregation and alter cargo abundance (Ahmadian et al. 2024; Gelibter et al. 2022), only indirect evidence has suggested that RNA fragmentation in EVs might occur during freezing. To date, there is a lack of direct transcript‐length profiling directly comparing fresh versus frozen EV‐RNA. Therefore, to our knowledge, the present study's observation that freezing alters transcript‐size distribution and increases the proportion of incomplete isoforms (ISM) provides one of the first direct lines of evidence that preprocessing can bias long‐RNA representation in EV preparations, representing a novel and valuable contribution to the field.

At the gene level, reads assigned to mRNA gene entries mapped 10,230 protein‐coding genes, with the 50 most abundant representing ∼ 60% of total mRNA reads, with ∼95% of these (47/50) consistent across Fresh and Frozen samples. Among the 302 identified lncRNAs, 2 accounted for almost 90% of the lncRNA reads in both conditions. These results highlight that highly abundant transcripts are reliably recovered regardless of pre‐processing treatment and global expression patterns and the most abundant transcripts remain stable.

Overall, these findings demonstrate that pre‐processing treatment can influence the representation of long transcripts in EV‐RNA. Freezing generally preserves the overall RNA profile and the core EV cargo, but long and low‐abundance transcripts are more susceptible to degradation, potentially impacting analyses that rely on rare or structurally fragile RNAs, such as lowly expressed lncRNAs or full‐length mRNAs. For general profiling of abundant EV RNA species, short‐term freezing at −20°C is unlikely to pose major problems; however, for sensitive downstream applications, storage at −80°C and careful handling are recommended to minimize subtle biases based on previous literature.

### 4.4 Functional Insights Into Predominant Long‐RNAs EV‐Associated Transcripts

The most abundant transcripts identified within *Sparus aurata* plasma‐derived EVs provide insights into the potential biological roles of circulating vesicles in this species. EVs are released by nearly every cell type and able to transfer diverse molecules (including different RNA biotypes) from donor to recipient cells. While most studies have focused on short RNAs such as miRNAs, the presence and potential roles of longer transcripts, including mRNAs and lncRNAs, remain less well understood (O'Grady et al. 2022).

The vast majority (85%) of the top 50 protein‐coding genes corresponded to ribosomal proteins and ribosome‐associated factors, indicating a strong enrichment of components related to protein synthesis and translation regulation. Such enrichment has been reported in EVs from different cell types and across different mammals (e.g., bovine follicular EVs, porcine oviduct EVs, human plasma EVs) (Almiñana et al. 2018; Fabbiano et al. 2020; O'Grady et al. 2022; Statello et al. 2018), suggesting selective packaging of ribosomal transcripts involved in maintaining translational capacity or regulating protein turnover in recipient cells. Besides, it has been suggested that ribosomal proteins (RPs) and their transcripts in EVs may play additional roles in modulating responses in recipient cells (Fabbiano et al. 2020). These findings may suggest that the enrichment of ribosomal mRNAs in EVs is an evolutionarily conserved phenomenon across vertebrates and could be associated with processes of intercellular communication.

Analysis of the long fraction of the poly‐adenylated EV transcriptome showed a clear enrichment of genes involved in core cellular functions, including protein synthesis, energy metabolism, hematopoiesis, and angiogenesis, patterns or functions previously reported in EV cargo (Abdelgawad et al. 2025; Kraft et al. 2025; O'Grady et al. 2022; Prieto‐Vila et al. 2021). The abundance of ribosomal and metabolic mRNAs suggests that these EVs may help to coordinate metabolic balance and energy regulation, which is particularly relevant in the variable environmental and nutritional conditions of aquaculture. Overall, these findings support the view that fish plasma EVs mirror key aspects of cellular physiology and act as active mediators of communication and adaptation, highlighting their potential role in organismal homeostasis and responses to environmental stress.

A total of 302 distinct lncRNA transcripts were found, demonstrating the suitability of the selected protocol for lncRNA detection. Most of these lncRNAs are computationally predicted and lack functional annotation, which prevented functional enrichment analysis at the gene level. Interestingly, two lncRNAs accounted for almost 90% of the lncRNA reads, highlighting a highly skewed abundance distribution (which might have been amplified by sequencing). Characterizing the structure and interaction of lncRNAs is crucial to understand their mechanisms in cells and EVs (Qian et al. 2019; L. Wang et al. 2025; Y. Wang et al. 2025). LncRNAs can regulate gene expression through many mechanisms, such as transcriptional regulation, translation regulation, protein modification, and RNA‐protein complex formation (Fatima and Nawaz 2017; Peña‐Flores et al. 2024; Statello et al. 2018).

To explore their potential functions within EVs, we investigated interactions between the two most abundant lncRNAs (*LOC115578803* and *LOC115584152*) and the pool of mRNA transcripts contained in the EVs. *LOC115584152* was predicted to interact with 16 mRNAs. They included several genes involved in mitochondrial function and oxidative phosphorylation (e.g. ND6, cytochrome c oxidase subunit 8A, and mitochondrial ribosomal protein L41), and also genes involved in transcriptional regulation and chromatin dynamics, such as Mediator complex subunit 9 and histone variants (H2B 1/2‐like, H2A.L3). Other genes from this list included P2Y purinoceptor 1‐like and GTP‐binding protein Rhes‐like, which points to intricate intracellular and extracellular signaling pathways. The presence of hemoglobin subunit alpha‐1, Otospiralin 2, and Dicentracin‐like also indicates specialized functional roles in oxygen transport, sensory processes, and immune defense, respectively. Overall, the set of predicted interactions points towards *LOC115584152* participating in the regulation of metabolic activity, dynamic gene expression, and tissue‐specific processes within EVs.

*LOC115578803* was predicted to interact with 94 mRNAs. Interestingly, 75% of the mRNA that potentially interact with *LOC115584152* (12/16) also interact with *LOC115578803*. The set of mRNAs interacting with *LOC115578803* included transcription factors, mitochondrial and ribosomal components, immune‐related genes, metabolic enzymes, and structural proteins. The presence of multiple transcriptional regulators including RNA polymerase II subunit J, Mediator complex components, and general transcription factors (TFIIH subunit 5), along with several bHLH transcription factors such as Hes1, Hes4, and Twist1b, suggests that the lncRNA could influence gene expression programs and developmental pathways, by potentially modulating transcriptional machinery or chromatin accessibility. The presence of multiple histone variants (H2A‐like, H2B 1/2‐like, H2A.X, and H2A.L3) supports this notion, suggesting involvement in epigenetic regulation or chromatin remodeling. On the other hand, the enrichment of mitochondrial and respiratory genes including cytochrome c oxidase subunit 8A, NADH dehydrogenase subunit A3, MICOS complex subunit MIC26, and malate dehydrogenase 2, may suggest a connection between the lncRNA and energy metabolism or redox homeostasis, possibly affecting cellular responses to stress or differentiation. The presence of cell cycle regulators (such as G_0_/G_1_ switch proteins, growth arrest and DNA damage–inducible beta) implies a role in proliferation control. The multiple occurrences of polyubiquitin‐like and ubiquitin‐related enzymes point to proteostasis and protein turnover regulation, crucial for maintaining cellular homeostasis. Meanwhile, ribosomal and translation‐related genes (e.g., elongation factor 1 alpha, or ribosomal proteins S27/S28) emphasize the implication in the regulation of protein synthesis. The gene set also features immune and defense‐associated genes, such as pleurocidin‐like peptides, dicentracin‐like, macrophage migration inhibitory factor‐like, or C‐C and C‐X‐C motif chemokines, which may also suggest that *LOC115578803* may participate in intercellular immune signaling, mediated through EVs in teleost plasma.

Overall, the mRNA potentially interacting with the EV‐contained lncRNAs suggests that these lncRNAs may play a multifaceted regulatory role in cellular communication, transcriptional control, and metabolic adaptation. Thus, EV‐lncRNAs likely act as a regulatory hub, highlighting their potential importance in EV‐mediated intercellular signaling and cellular reprogramming.

Because lncRNAs often exert their functions through interactions with RBPs, we explored potential associations between the two most abundant lncRNAs identified in *Sparus aurata* EVs and the RNPs encoded by the most highly represented EV‐mRNAs in our study. Remarkably, 40 of the proteins encoded by these mRNAs were predicted to interact with one or both lncRNAs, suggesting that these vesicles may carry preassembled or functionally related RNA–protein modules. Co‐packaging of lncRNAs and their potential binding partners is increasingly recognized, and may reflect coordinated export mechanisms, or at least imply that these lncRNAs contribute to post‐transcriptional regulation, ribosome assembly, or translational control in recipient cells (Fatima and Nawaz 2017; Statello et al. 2021). This finding supports the emerging view that EVs act not only as carriers of RNA and protein separately but may also contribute to coordinated RNA cargo delivery involved in intercellular communication. To our knowledge, while lncRNA–RBP co‐packaging has been reported in mammalian systems (Fabbiano et al. 2020), it has not been reported in fish. Although RBP content or proteomic analysis was not performed in this study, we speculate that the co‐packing of abundant EV‐mRNAs encoding proteins with the potential to bind abundant EV‐lncRNAs may have functional implications once translated in the recipient cells.

Research on EV‐lncRNAs has so far been dominated by studies in cancer biology, particularly in the context of biomarker discovery and signaling modulation (Fatima and Nawaz 2017; Marima et al. 2024; Nie et al. 2021; Peña‐Flores et al. 2024). In contrast, there has been little in‐depth mechanistic research in aquatic animals (Zhao, Deng et al. 2021). Given the still limited annotation of teleost lncRNAs, our results provide a methodological and conceptual framework for exploring their biological roles through integrative analyses of EV‐associated mRNAs and lncRNAs. Nonetheless, lncRNA discovery and annotation will require de novo assembly, multi‐tool coding‐potential filtering, as well as empirical validation studies.

### 4.5 Study Limitations and Future Directions

Despite providing new insights into the fish EV‐RNA landscape, more specifically in *Sparus aurata*, several limitations should be acknowledged. First, the sample size and low RNA input intrinsic to plasma‐derived EVs in fish may have limited the detection of low‐abundance transcripts and introduced stochastic variation in sequencing coverage. Second, although nanopore sequencing enables full‐length transcript detection, the use of poly(A)‐dependent library preparation is biased toward polyadenylated RNAs, overlooking non‐polyadenylated or highly structured RNA species, such as certain lncRNAs. Third, functional predictions of lncRNA–mRNA and lncRNA–protein interactions were based on in silico analyses and require experimental validation. Fourth, while TEM, DLS and RNA profiling confirmed the vesicular nature of the isolates, potential co‐isolation of lipoprotein‐ or AGO‐associated RNA cannot be entirely excluded, as has been reported in column‐based EV isolation methods. Fifth, the comparison between exoRNeasy Midi and Maxi kit formats was performed using different biological units (individual versus pooled samples). Consequently, potential effects of input volume cannot be completely separated from biological variability. Future studies should evaluate volume‐dependent extraction efficiency by processing aliquots of the same biological sample or pool in parallel using different input volumes and kit formats. Finally, annotation limitations in the *Sparus aurata* genome may have restricted accurate classification of some long transcripts and non‐coding RNAs.

Future studies combining orthogonal EV isolation strategies, higher sequencing depth, and functional validation experiments will be necessary to confirm the biological relevance of these findings and to elucidate the mechanistic roles of EV‐associated long RNAs in fish physiology.

## 5 Conclusion

Overall, the EV‐RNA cargo appeared largely conserved between fresh and frozen plasma samples, with comparable RNA biotype composition and high overlap among the most abundant transcripts. This indicates that freezing does not substantially alter the overall representation of EV‐RNAs. However, more subtle differences were observed at the transcript level, particularly in size distribution and isoform completeness, with frozen samples showing a higher proportion of incomplete isoforms and a relative enrichment in shorter RNA species. Therefore, while freezing provides an acceptable strategy for EV‐RNA preservation and overall transcriptome profiling, good for biomarker screening of abundant RNAs or for detecting major differences across treatments, it may introduce small biases that become significant in specific contexts such as in studies focusing on low‐abundance long transcripts (e.g., lncRNAs, full‐length mRNAs) or Isoform‐level analyses, where degradation may skew the ratio of full‐length vs incomplete isoforms. These results emphasize the need for consistent preprocessing and careful control of storage conditions in comparative EV‐RNA studies.

Finally, this study not only confirmed the presence of lncRNAs in *S. aurata* EVs, identifying 302 distinct transcripts, and demonstrating the suitability of the selected protocol for lncRNA detection, but also provides a framework for exploring the potential biological function of unannotated lncRNAs by assessing their potential functions through predicted interactions with mRNAs and their codified RNPs within EVs.

## Author Contributions

**Antonio Espuela‐ortiz**: methodology, data curation, investigation, formal analysis, Writing – original draft, writing – review and editing. **Estrella Antón‐gonzález**: investigation, formal analysis, writing – review and editing. **Louis Bonnefous**: formal analysis, writing – review and editing, writing – original draft. **M. Virginia Martín**: resources, writing – review and editing. **Salvador Jeréz**: resources, writing – review and editing. **Deiene Rodríguez‐barreto**: conceptualization, methodology, data curation, investigation, formal analysis, supervision, funding acquisition, project administration, resources, writing – original draft, writing – review and editing.

## Funding

This work has been funded by the European Union. Views and opinions expressed are however those of the author(s) only and do not necessarily reflect those of the European Union or the European Research Council Executive Agency. Neither the European Union nor the granting authority can be held responsible for them. This work is supported by ERC grant (EPIAQUA, 101076685https://doi.org/10.3030/101076685).

## Conflicts of Interest

The authors declare no conflicts of interest.

## Supporting information




**Supporting Information**: jex270175‐sup‐0001‐FiguresS1‐S3.pdf

**Supporting Information**: jex270175‐sup‐0002‐FigureS4.pdf

**Supporting Information**: jex270175‐sup‐0003‐FigureS5.pdf

**Supporting Information**: jex270175‐sup‐0004‐FigureS6.pdf

**Table S1**: Sample metadata and RNA metrics, including ID, specimen size and weight, plasma volume, kit format and pre‐processing, RNA concentration and yield, and fragment distribution based on TapeStation electropherograms.
**Table S2**: RNA‐seq gene and read counts by RNA biotype (mRNA, lncRNA, sncRNA, other RNA) for reference genome, fresh, and frozen samples, including total and unannotated reads, and biotype ratios.
**Table S3**: Genomic features and RNA‐seq read counts per gene across fresh and frozen samples, including coordinates, feature type, gene IDs, lengths, read counts, relative abundances, and annotations.
**Table S4**: Enrichment analysis results of the full list of mRNAs showing ontology terms, parent terms, and FDR‐adjusted p‐values across Fresh and Frozen pools and Fresh subsamples.
**Table S5**: Statistical test results for RNA integrity and quantification metrics across kit formats and pre‐processing treatments.
**Table S6**: Isoforms identification per sample.
**Table S7**: Summary of GO term clusters based on semantic similarity and GO hierarchy. Each cluster lists preprocessing treatment, cluster number, the number of GO terms within each cluster, representative GO ID and name, cumulative significance (∑‐log_10_(p_adj_)), and Parent Go terms included in each cluster.
**Table S8**: Predicted RNA–RNA interactions including between LOC115584152 and potential target mRNAs from EVs transcriptome including sequence coordinates, hybridization energies, interaction lengths, and functional annotations of target mRNAs.
**Table S9**: Predicted RNA–RNA interactions between *LOC115578803* and potential target mRNAs from EVs transcriptome including sequence coordinates, hybridization energies, interaction lengths, and functional annotations of target mRNAs.
**Table S10**: Predicted RNA–protein interactions of *LOC115584152* with RNP codify by top mRNAs with key sequence, charge, and accessibility features supporting binding confidence.
**Table S11**: Predicted RNA–protein interactions between LOC115578803 and RNP encoded by top mRNAs.

## Data Availability

All data are available in the main text, supporting information tables or (to be) deposited in an appropriate repository. The data that support the findings of this study are available from the corresponding author upon reasonable request.
