# Supplementary material for: Evaluating Plasma‐Handling Effects on Extracellular Vesicle RNA Yield and Cargo Profiles in Fish: A Methodological Insight
Source: J Extracell Biol. 2026 Oct 15;5(10):e70175. doi: 10.1002/jex2.70175 (PMC13670421; doi:10.1002/jex2.70175)
Supplement: Supplementary file 1 [file JEX2-5-e70175-s001.pdf]

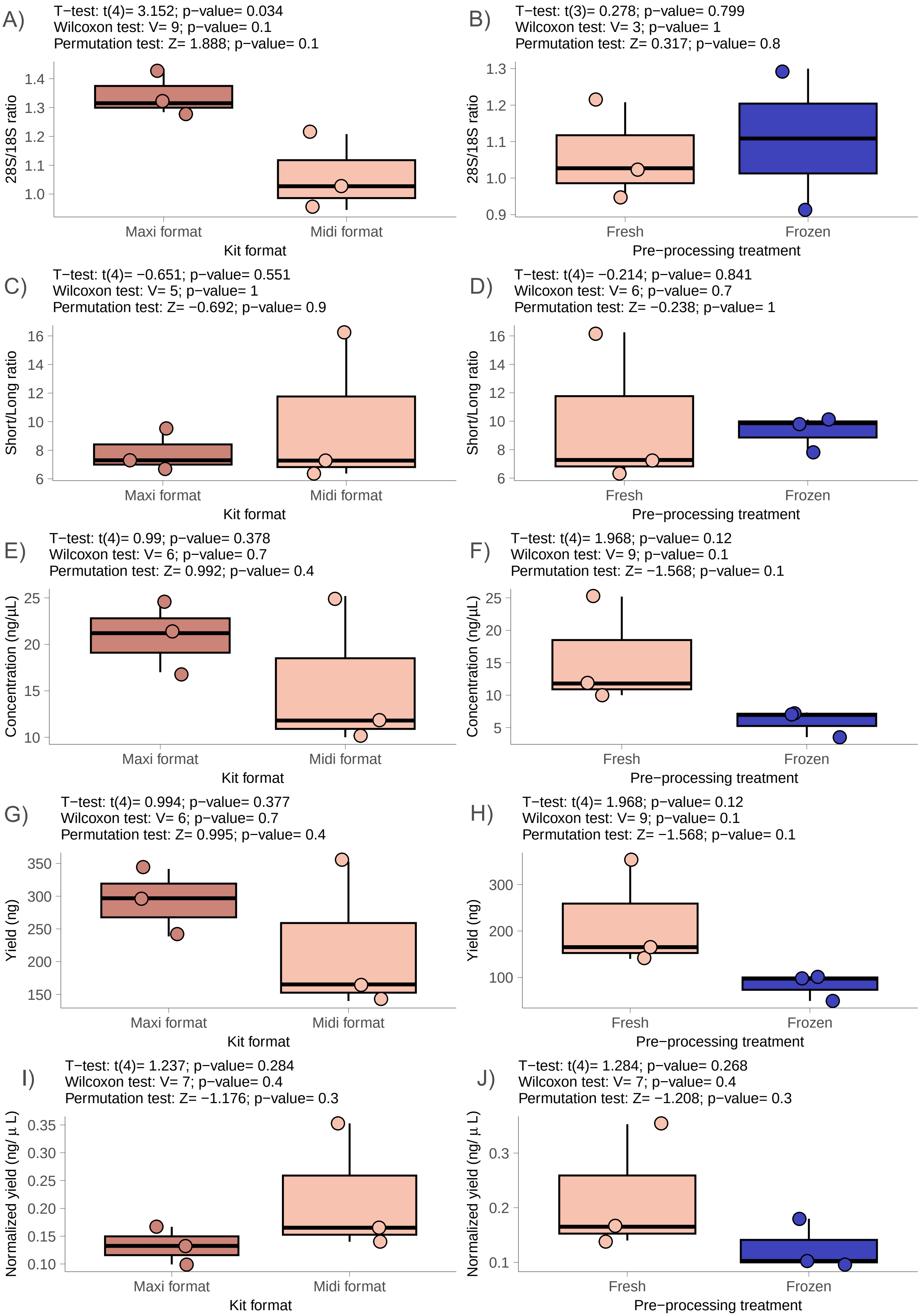

Figure S4. RNA integrity and quantification metrics comparing kit formats (Maxi [~2 mL] vs. Midi [~1 mL]) (panels A, C, E, G, and I) and pre-processing treatments (Fresh [samples processed right after collection] vs. Frozen [samples processed after two weeks of storage at -20 ºC]) (panels B, D, F, H, and J). Boxplot for 28S/ 18S ratio (panels A and B), Short/ Long ratio (panels C and D), RNA concentration (ng/µL) (panels E and F), RNA yield (ng) (panels G and H), RNA normalized yield by input volume (ng/µL). Dark clay color represents Fresh samples processed using the Maxi format, light clay color represents Fresh samples processed using the Midi format, and blue purple blue color represents samples processed using the Midi format after being frozen for two weeks.
