# Supplementary material for: Evaluating Plasma‐Handling Effects on Extracellular Vesicle RNA Yield and Cargo Profiles in Fish: A Methodological Insight
Source: J Extracell Biol. 2026 Oct 15;5(10):e70175. doi: 10.1002/jex2.70175 (PMC13670421; doi:10.1002/jex2.70175)
Supplement: Supplementary file 2 [file JEX2-5-e70175-s002.pdf]

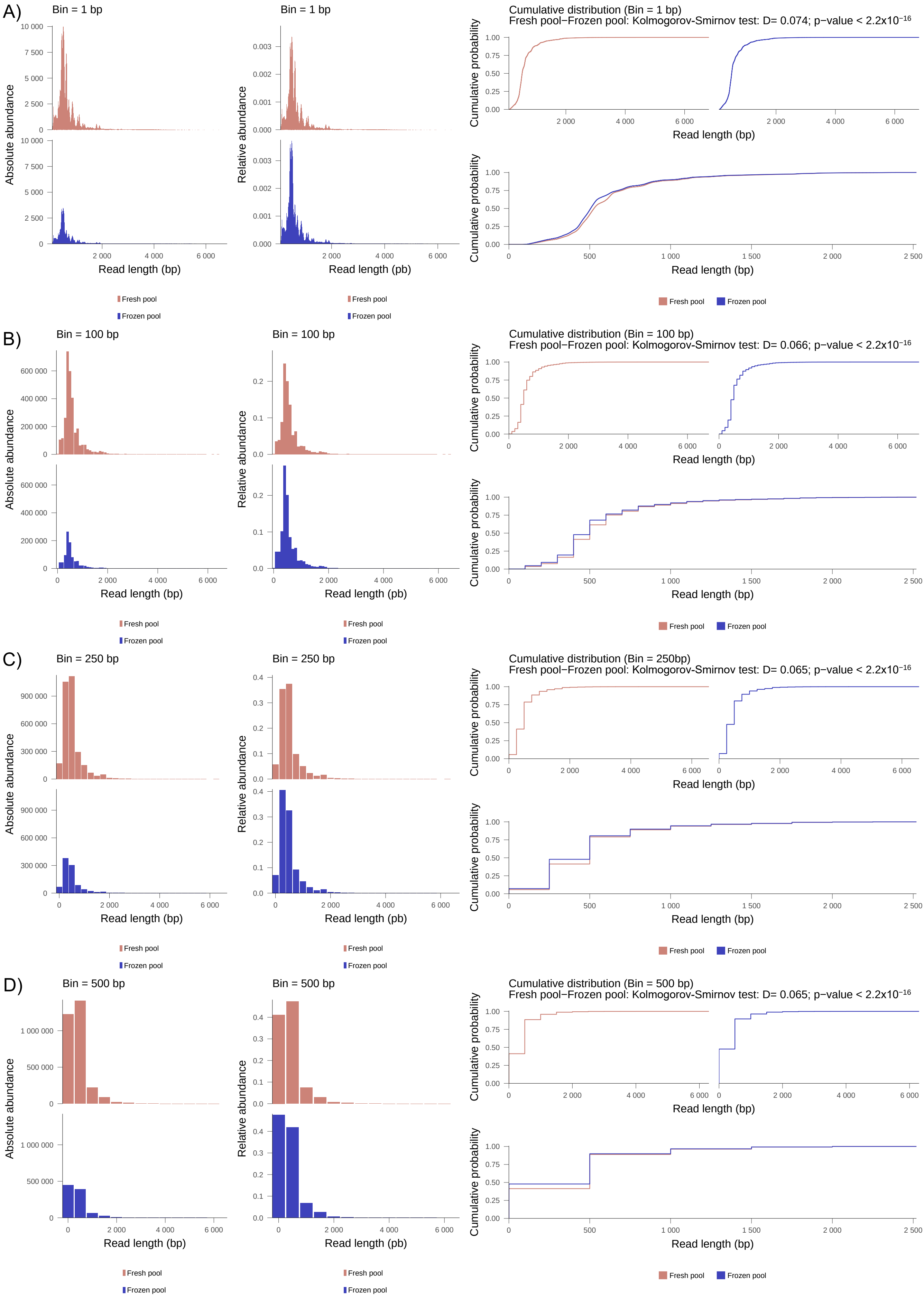

Figure S5. Transcript length distribution comparison between Fresh and Frozen pools across different bin sizes. Bin sizes 1 bp (panel A), 100 bp (panel B), 250 bp (panel C), and 500 bp (panel D). For each bin size there are four plots: Histograms of transcript length distribution per pool in absolute and relative abundance (first and second columns, respectively), full cumulative distribution of transcript length per pool (third column, upper half), and zoomed-in view highlighting differences between transcript length distributions (third column, lower half).

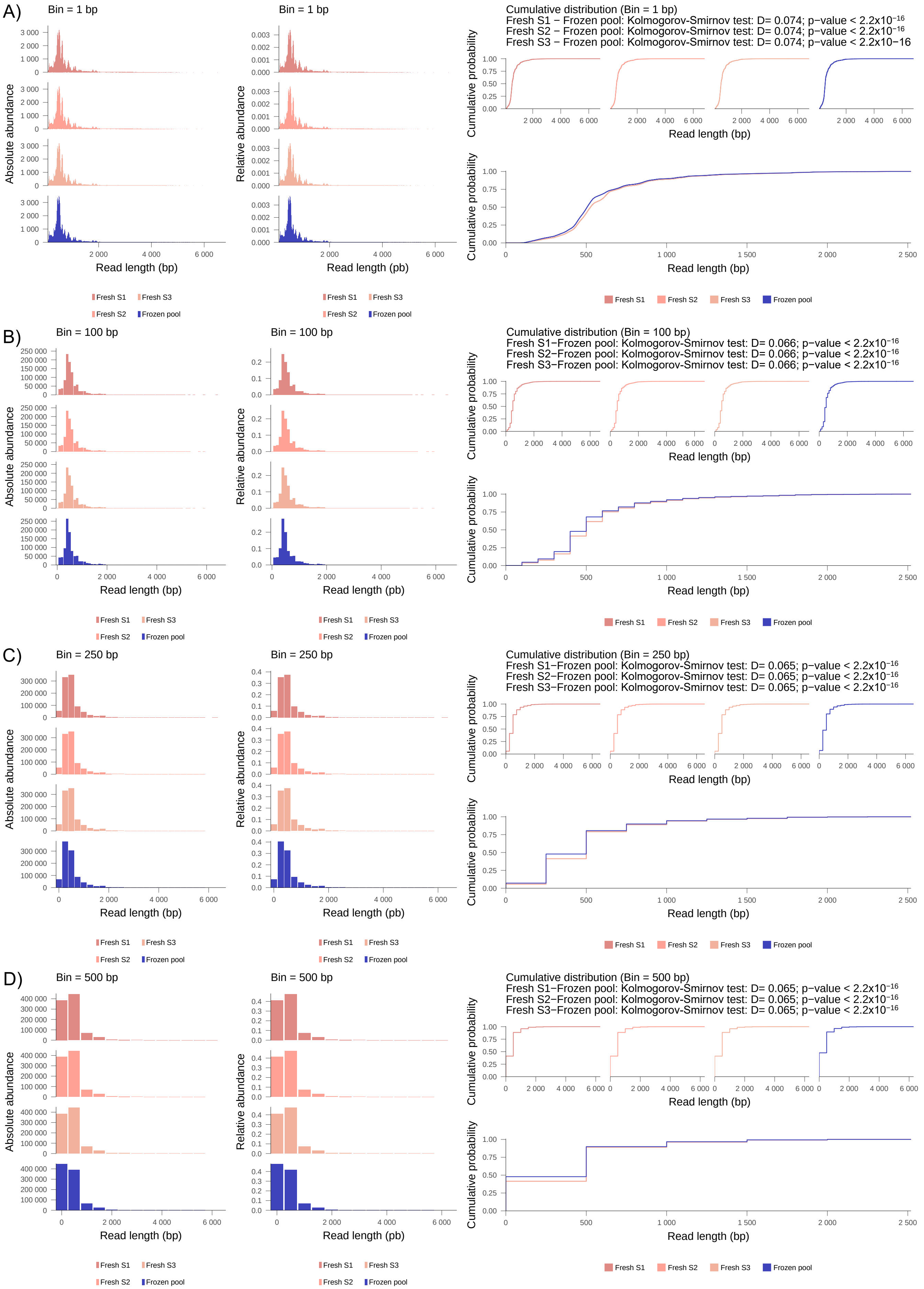

Figure S5-bis. Transcript length distribution comparison between Fresh subsamples and Frozen pool across different bin sizes. Bin sizes 1 bp (panel A), 100 bp (panel B), 250 bp (panel C), and 500 bp (panel D). For each bin size there are four plots: Histograms of transcript length distribution per sample in absolute and relative abundance (first and second columns, respectively), full cumulative distribution of transcript length per sample (third column, upper half), and zoomed-in view highlighting differences among transcript length distributions (third column, lower half).
