# Supplementary material for: Evaluating Plasma‐Handling Effects on Extracellular Vesicle RNA Yield and Cargo Profiles in Fish: A Methodological Insight
Source: J Extracell Biol. 2026 Oct 15;5(10):e70175. doi: 10.1002/jex2.70175 (PMC13670421; doi:10.1002/jex2.70175)
Supplement: Supplementary file 3 [file JEX2-5-e70175-s004.pdf]

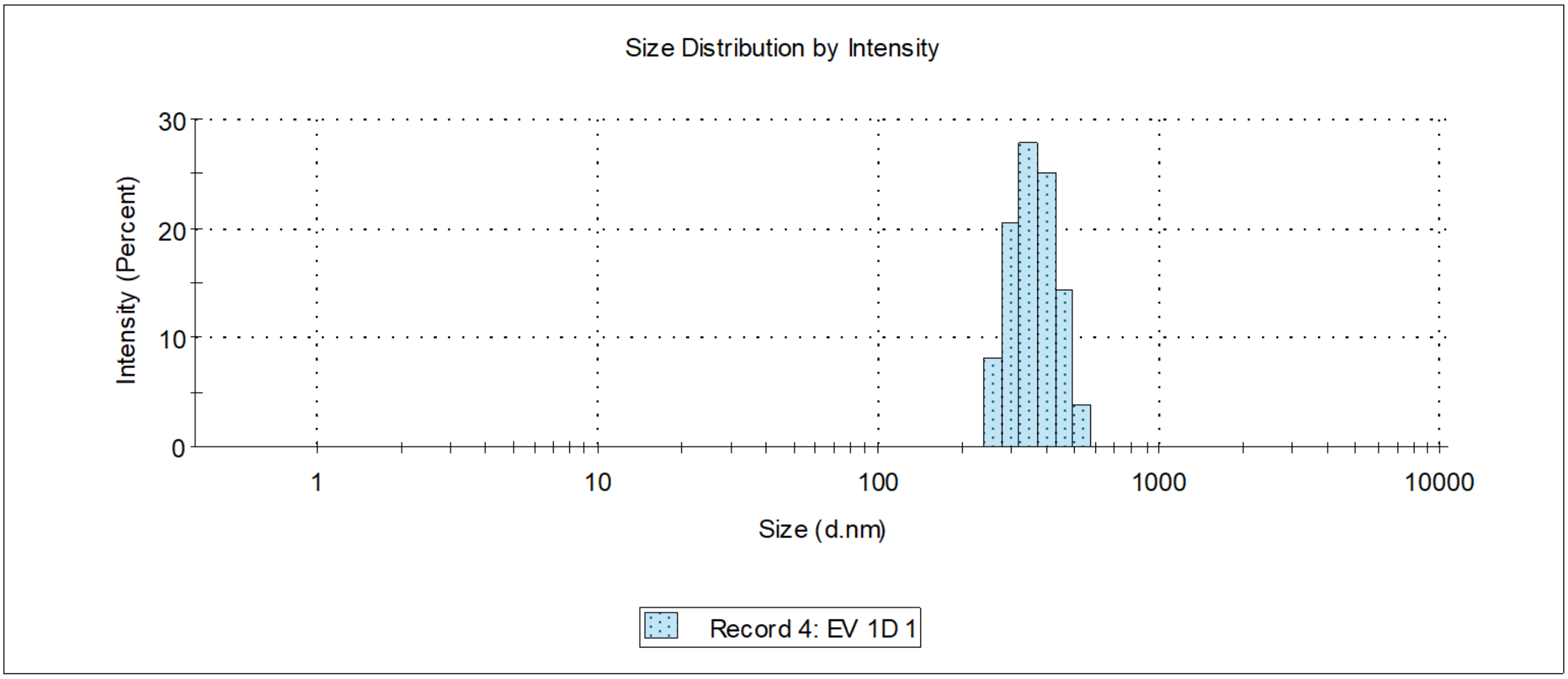


Figure S1. Size distribution of Fresh plasma-derived structures compatible with extracellular vesicles (EVs) from dynamic light scattering (DLS) analyses performed on a Zetasizer Nano ZS (Malvern) equipped with a HeNe 633 nm laser and operating on a 173 nm angle. The x-axis represents particle size (nm) and the y-axis represents intensity (%). The detected structures show a mean size of approximately 300-400 nm, indicating a slightly heterogeneous population.

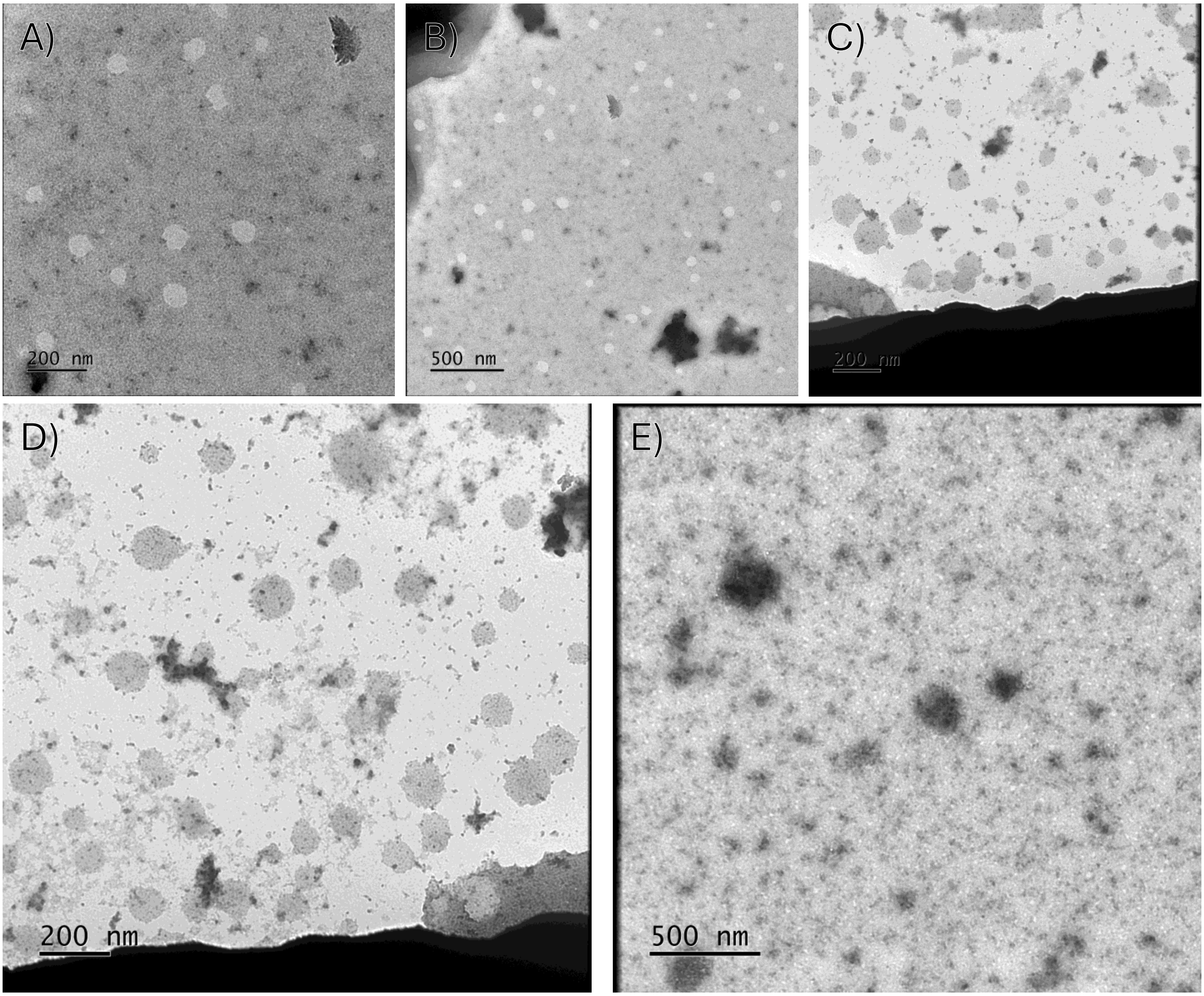


Figure S2 . Representative transmission electron micrographs showing structures compatible with extracellular vesicles (EVs) isolated from *Sparus aurata* plasma stored at −20 °C for two weeks ("Frozen"). Samples were negative stained with uranyl acetate and imaged using a JEM-1400Flash transmission electron microscope. Panels A–D: sample fixed with 2.5% glutaraldehyde; Panel E: sample fixed by freezing at −20 °C. Black horizontal lines in each panel represent a reference scale corresponding to the magnification used during image acquisition.

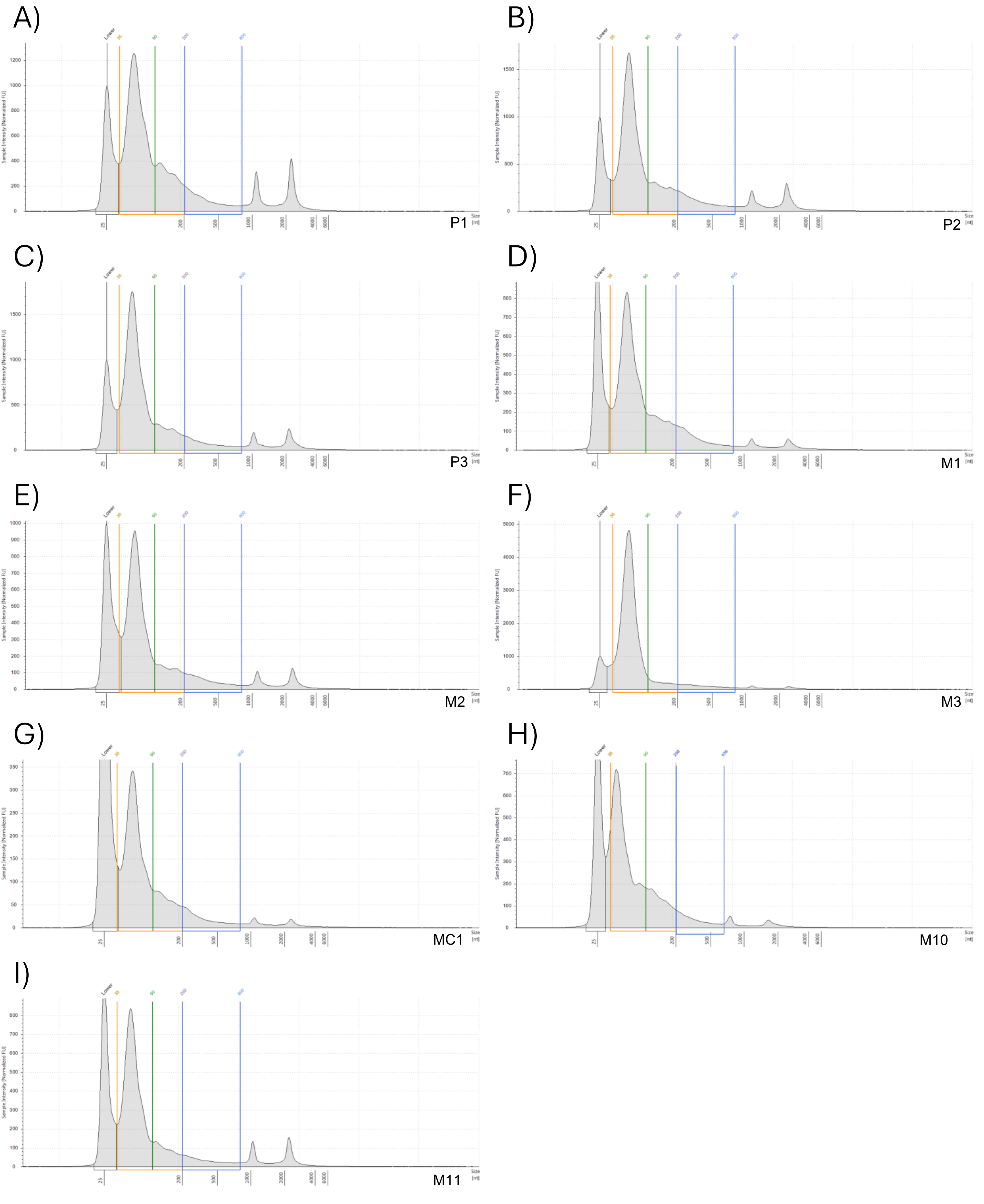


Figure S3. RNA quality control from TapeStation (TM) displaying electrophoretic profiles representing size distribution of transcripts per sample. Panels A-C: Maxi format Fresh samples; panels D-F: Midi format Fresh samples; panels G-I: Midi format Frozen samples. Orange lines represent the lower and upper thresholds for short transcripts (35-200 bp) to exclude the intrinsic calibration marker, green lines represent the 90 bp threshold, and blue lines represent the lower and upper thresholds of the long transcripts (>200 bp)
