# Supplementary material for: Evaluating Plasma‐Handling Effects on Extracellular Vesicle RNA Yield and Cargo Profiles in Fish: A Methodological Insight
Source: J Extracell Biol. 2026 Oct 15;5(10):e70175. doi: 10.1002/jex2.70175 (PMC13670421; doi:10.1002/jex2.70175)
Supplement: Supplementary file 4 [file JEX2-5-e70175-s005.pdf]

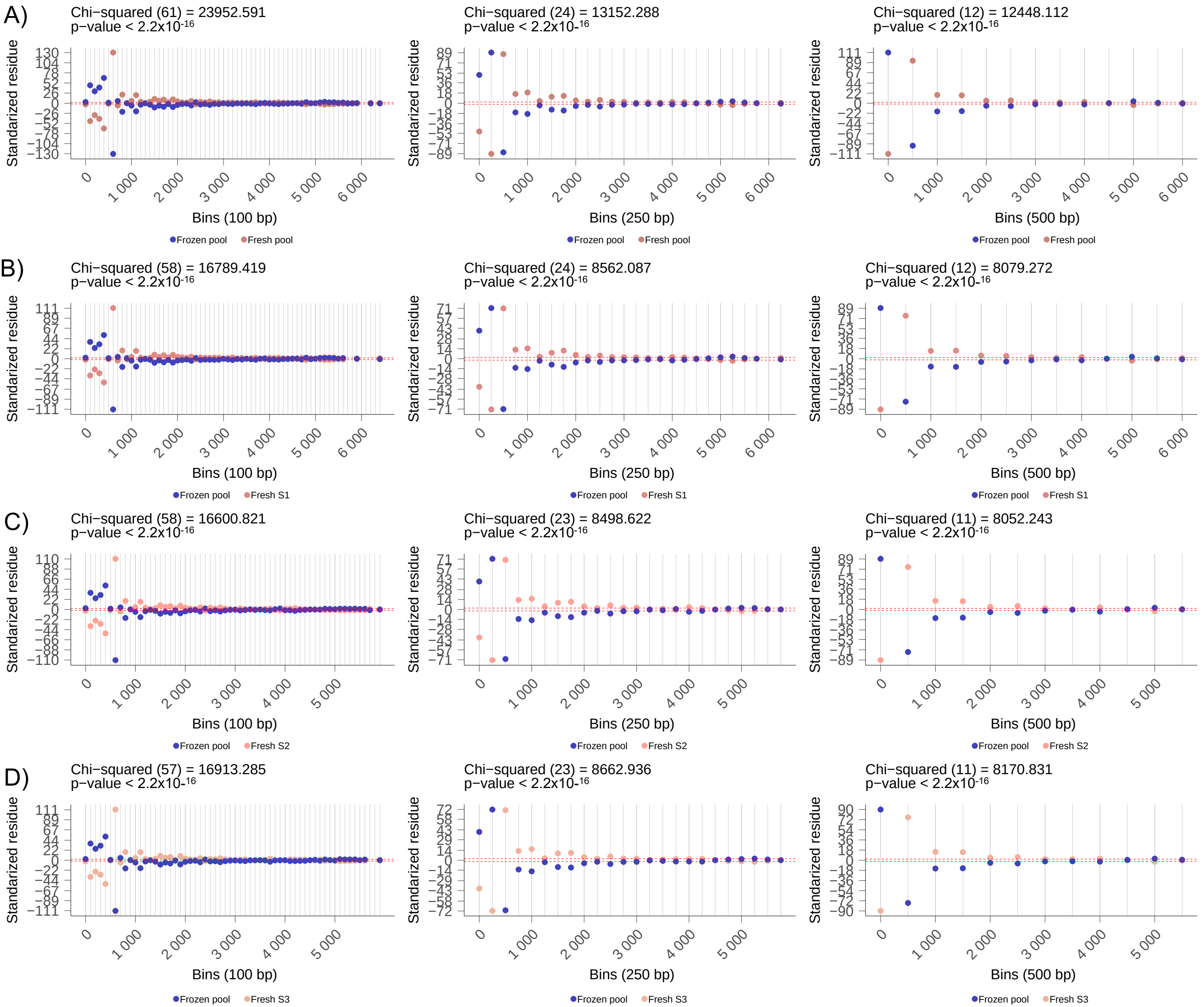

Figure S6. Chi-squared standardized residues from transcript length distribution comparisons between Fresh samples and Frozen pool. Comparison between Fresh and Frozen pools (panel A), Fresh Subsample 1 (Fresh S1) and Frozen pool (panel B), Fresh Subsample 2 (Fresh S2) and Frozen pool (panel C), Fresh Subsample 3 (Fresh S3) and Frozen pool (panel D). Each panel displays the results for three different bin sizes (from left to right: 100, 250, and 500 bp). Across all comparisons, a consistent over-representation of short transcripts (100 - 400 bp) was observed in the Frozen pool.
